# Prognostic Value of Preoperative Assessment of Left Ventricular Function in Patients Undergoing Percutaneous Coronary Intervention

**DOI:** 10.31083/j.rcm2403080

**Published:** 2023-03-06

**Authors:** Ming Yang, Fan Guo, Yin-Jian Yang, Zhi-Cheng Jing, Kai Sun

**Affiliations:** ^1^Department of Cardiology, State Key Laboratory of Complex Severe and Rare Diseases, Peking Union Medical College Hospital, Chinese Academy of Medical Sciences and Peking Union Medical College, 100730 Beijing, China

**Keywords:** percutaneous coronary intervention, left ventricular ejection fraction, prognostic

## Abstract

**Background:**

Patients may experience a decline in cardiac function even after successful percutaneous coronary intervention (PCI). It is apparent that the assessment of left ventricular (LV) function before PCI is often overlooked. The purpose of this review is to explore the significance of LV function assessment before PCI by comparing the differences in short- and long-term PCI outcomes between patients with different LV ejection fraction (LVEF) stratified preoperatively.

**Methods:**

PubMed and Scopus were searched to identify potential studies from January 1, 2001 through January 1, 2022.

**Results:**

A total of 969,868 participants in 33 studies at different stratifications of baseline LVEF were included in this review and their PCI outcomes were stratified for analysis. The hazard ratio of all-cause mortality within 30 days, one year and greater than 1 year after PCI between patients with abnormal and normal LVEF were 2.96 [95% CI, 2.2, 3.98], 3.14 [95% CI, 1.64, 6.01] and 3.08 [95% CI, 2.6, 3.64]; moderately impaired LV function versus normal were 2.32 [95% CI, 1.85, 2.91], 2.04 [95% CI, 1.37, 3.03], 1.93 [95% CI, 1.54, 2.44]; poor LV function versus normal were 4.84 [95% CI, 3.83, 6.1], 4.48 [95% CI, 1.37, 14.68], 6.59 [95% CI, 4.23, 10.27].

**Conclusions:**

A moderate or severe reduction in patients’ LVEF may have a serious impact on PCI prognosis. We strongly advocate for adequate assessment of LVEF before PCI as this will assist in choosing the optimal revascularization and postoperative treatment, thereby reducing short- and long-term mortality.

## 1. Introduction

Since percutaneous coronary intervention (PCI) was introduced in 1977 [1], important advances have been made. Early and long-term outcomes of PCI have been improved with the advent of lower profile balloons, bare-metal stent (BMS), drug eluting stent (DES), improved guide-wire support, increased use of adjuvant drugs and hemodynamic support devices. Recent studies have shown that increased use of PCI reperfusion has led to a decrease in acute coronary syndrome (ACS) mortality. In patients with ST segment elevation myocardial infarction (STEMI), primary PCI can limit infarct size and preserve left ventricular (LV) systolic function [2, 3]. Despite being highly effective in reducing the need for repeat revascularisation compared with BMS, early-generation DES were associated with an increased risk of late (>1 year) thrombotic events due to an excess of stent thrombosis [4, 5, 6]. Currently, new-generation DES feature lower antiproliferative drug loads, thinner stent metallic struts and more biocompatible durable or biodegradable polymers than previous devices [7, 8].

PCI is a mature technology that is highly utilized in clinical practice. Lack of evaluation of the LV function before PCI may result in the failure to select the optimal revascularization protocol. In a 2021 network meta-analysis by Yujiro Yokoyama *et al*. [9], coronary-artery bypass grafting (CABG) remained the treatment of choice in patients with coronary artery disease and low LV ejection fraction (LVEF). Studies have shown that approximately one-third of patients who undergo PCI [10, 11, 12] suffered from LV dysfunction—an important predictor of post PCI death and major adverse cardiac events (MACE) [13, 14]. PCI does not improve or maintain cardiac function in all STEMI patients with data demonstrating that 4.7–8.6% of patients may experience a decline in cardiac function after successful primary PCI [15, 16]. Therefore, the stratification of LVEF risk assessment before PCI is particularly important but often overlooked. According to the audit of European Association for Percutaneous Cardiovascular Intervention in the UK, only 46% of patients undergoing PCI had ever received LV classification [10]. According to the Mayo Clinic, information on LV function is available in only 60% cases [17] with the main reason being that PCI is increasingly performed in the setting of ACS that requires timely intervention [18, 19]. Comprehensive clinical assessment is sacrificed for the sake of expediency, resulting in insufficient time to assess LV function before PCI. Congestive heart failure (CHF) after STEMI PCI is the primary reason behind the increase in morbidity and mortality [20]. Patients at high risk for CHF need to be identified to select more appropriate post infarction therapies. We believe that LV assessment is helpful for patient risk stratification, even in the context of ACS. This ensures the preoperative awareness of the high-risk nature of the surgery and facilitates the proper revascularization [21].

This study aims to explore the significance of LV function assessment before PCI by comparing the differences in short- and long-term PCI outcomes between patients with different LVEF levels stratified preoperatively along with raising the importance of the evaluation of LVEF before PCI.

## 2. Materials and Methods

The protocol was registered on INPLASY (INPLASY202220031) and is available on inplasy.com (https://doi.org/10.37766/inplasy2022.2.0031). Our systematic review was consistent with the PRISMA (Preferred Reporting Items for Systematic reviews and Meta-Analyses) statement [22].

### 2.1 Data Sources and Searches

PubMed and Scopus were searched to identify potential studies from January 1, 2001 through January 1, 2022 (**Supplementary Method M1**) There were no language restrictions. The reference list of previous systematic reviews [23, 24, 25, 26] was scrutinized.

### 2.2 Study Selection and Eligibility Criteria

We included observational studies or secondary analysis of intervention studies that reported prognosis of PCI. Outcomes of studies needed to be stratified according to LVEF. Two investigators performed title/abstract screening independently from each other. Following this, the full-text of potentially eligible studies was accessed by two investigators for determining eligibility and data extraction. Data evaluated included study design, age, gender, grouping rules, sample size, patients, country, follow-up periods, and study results. If the article did not provide data results, we used free software Engauge-digitizer (Version 12.1, Mark Mitchell, Baurzhan Muftakhidinov and Tobias Winchen *et al*.) [27] to obtain data from figures present [28]. We assessed study quality using items from the Newcastle-Ottawa Quality Assessment Scale (NOS) [29].

### 2.3 Outcome and Data Synthesis

The primary outcome was all-cause mortality stratified according to LVEF at baseline. The secondary outcomes were MACE and cardiac mortality in-hospital or long-term. We conducted random-effects or fix-effects meta-analysis of outcomes for which at least 2 studies contributed data. Categorical data were expressed as the pooled odds ratio (OR) or Hazard ratio (HR) with their 95% CIs using the inverse variance method. Heterogeneity was evaluated using both the χ^2^ test and the $I^{2}$ statistic. Publication bias was assessed using the Begg rank correlation test and the Egger weighted linear regression test for implementation strategies with at least 10 studies. All statistical tests were two sided and used a significance level of *p *< 0.05. We used STATA 15 (StataCorp, College Station, TX, USA) for all statistical analyses.

### 2.4 Subgroup Analysis

We analyzed three subgroups. (1) Patients with heart failure (New York Heart Association or Killip class >1) at baseline, heart failure with reduced ejection fraction (HFrEF) versus heart failure with preserved ejection fraction (HFpEF). (2) Patients undergoing elective PCI for chronic total occlusion (CTO). (3) STEMI patients.

## 3. Results

### 3.1 Literature Search

**Fig. 1.**
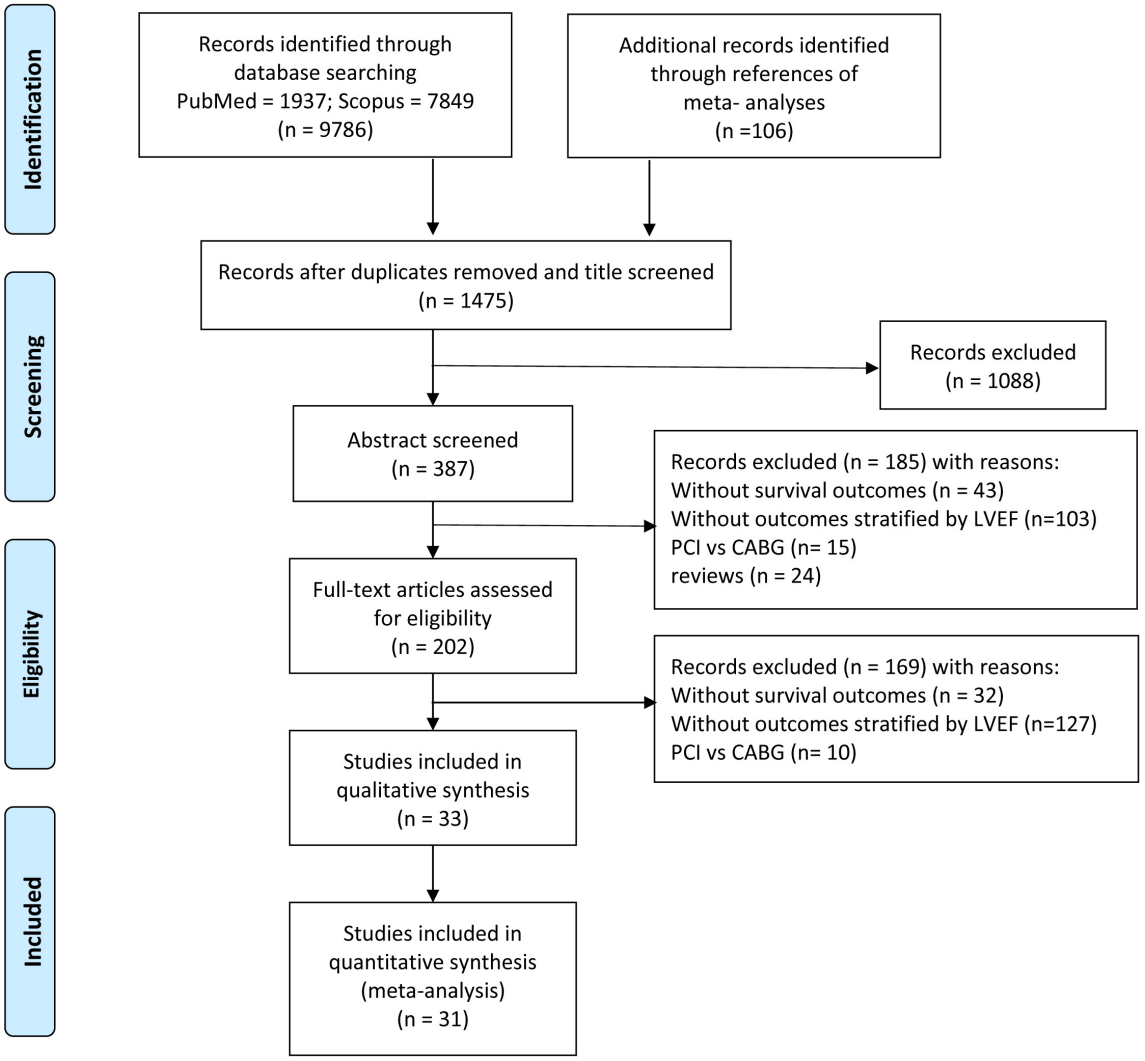
**Flowchart of study selection**.

We identified 9786 studies by database searching and 106 additional articles by reference tracking, of which 33 met inclusion criteria with resultant 969,868 patients. The flowchart of the article search and selection process is demonstrated in Fig. 1.

### 3.2 Study Characteristics

Of the 33 studies included, two were secondary analyses of randomized controlled trials [13] with the remaining 31 being observational studies (12 prospective and 19 retrospective). Participants in 6 studies [13, 14, 30, 31, 32, 33] were exclusively patients with STEMI, in 3 studies [34, 35, 36] participants were exclusively patients with CTO, and 3 studies [31, 37, 38] were patients with baseline heart failure. Thirteen studies [11, 30, 31, 32, 37, 38, 39, 40, 41, 42, 43, 44, 45] reported the prognostic outcomes during hospitalization, 14 studies [14, 33, 34, 35, 46, 47, 48, 49, 50, 51, 52, 53, 54, 55] reported the prognostic outcomes for greater than or equal to one year, and 6 studies [10, 13, 36, 56, 57, 58] reported the prognostic outcomes in both short and long term. The characteristics of the included studies are detailed (Table 1, Ref. [10, 11, 13, 14, 30, 31, 32, 33, 34, 35, 36, 37, 38, 39, 40, 41, 42, 43, 44, 45, 46, 47, 48, 49, 50, 51, 52, 53, 54, 55, 56, 57, 58]). The average NOS score of all included studies was 7.6 points, with 2 studies having a minimum score of 5 [32, 34] and 3 studies having score of 6 [30, 40, 43] (**Supplementary Table 1**). Five studies [30, 32, 34, 40, 43] were of low quality because they had too small sample sizes to be representative of the average level of the community, and confounders were not well controlled during the compassrison process, resulting in low comparability.

**Table 1.** **Characteristics of included studies**.

Study	Year	Study design	Age	Male	stratified LVEF%	Sample size	Patients	Country	Follow up
Alidoosti [46]	2008	Prospective Observational	56.1	69.0%	<40, 41–49, ≥50	2030	Patients with low, intermediate and high ejection fraction	Iran	long-term
Banga [30]	2019	Retrospective Observational	61.8 (12.9)	74.3%	<50, ≥50	249	Patients with STEMI treated with primary PCI	USA	in-hospital
Daneault [13]	2013	Secondary analysis of open-label, randomized trial	60.3 (54.6–72.3)	69.3%	<40, ≥40	2430	Patients with STEMI treated with primary PCI	USA	3-year
Doshi [31]	2019	Retrospective Observational	65.6 (13.4)	65.0%	<50, ≥50	31,180	hospitalisations undergoing STEMI-PCI	USA	in-hospital
El Awady [34]	2020	Prospective Observational	61.1 (8.2)	76.0%	<40, 40–49, ≥50	75	patients undergoing CTO PCI	Egypt	6 months
Galassi [35]	2017	Prospective Observational			<35, 35–50, ≥50	839	patients undergoing elective PCI of CTOs	Italy	6 months
Holper [47]	2006	Prospective Observational	69.2	54.5%	<50, ≥50	4697	patients undergoing PCI	USA	1 year
Jiang [48]	2017	Prospective Observational			<50, ≥50	10,490	patients undergoing PCI	China	2 year
Jiang [37]	2019	Retrospective Observational	68.6 (12.1)	69.8%	<40, 40–50, ≥50	1270	hospitalised patients with AMI undergoing emergency PCI	China	in-hospital
Marui [49]	2014	Prospective Observational	69.7 (9.7)		<50, ≥50	1432	patients undergoing first myocardial revascularization	Japan	5 years
Sardi [56]	2012	Retrospective Observational	68.6 (11.7)	75.8%	<25, 25–40, 41–50, ≥50	5337	patients undergoing PCI	USA	1 year
Shiga [50]	2009	Prospective Observational	66 (12)	73.7%	≤30, 30–40, >40	4122	patients with AMI, who were discharged alive	Japan	5 years
Son [51]	2016	Retrospective Observational		66.8%	≤60, >60	319	patients who underwent successful PCI	Korea	1 year
Sutton [52]	2016	Retrospective Observational	78 (71–84)	57.3%	≤35, 35–45, 45–55, ≥55	82,558	patients who underwent successful PCI	USA	1 year
Toma [36]	2017	Prospective Observational	66 (11)	87.0%	≤40, >40	2002	patients undergoing elective CTO PCI	Germany	2 years
Vakili [32]	2014	Prospective Observational	63.5 (12.6)	65.2%	≤25, 25–50, ≥50	401	patients with STEMI who underwent primary angioplasty	Iran	in-hospital
Wang [38]	2017	Prospective Observational	64.20 ± 10.75	79.2%	<40, 40–50, ≥50	1647	patients who had HF, and undergoing PCI/CAG		in-hospital
Ye [39]	2018	Retrospective Observational	62.18 (10.31)	69.9%	<50, ≥50	1600	patients who have undergone PCI	China	in-hospital
Zhong [53]	2020	Prospective Observational	63.69 (8.10)	87.5%	<50, ≥50	301	patients who underwent successful PCI	China	1 year
Alaswad [40]	2018	Retrospective Observational	69.57 (11.29)	75.3%	≤35, >35	891	patients undergoing PCI	USA	in-hospital
Biondi-Zoccai [57]	2011	Retrospective Observational	74.2 (9.2)	76.1%	<30, 30–45, >45	975	patients undergoing PCI	Italy	median of 18.2 months
De Silva [10]	2012	Retrospective Observational	65.7 (57.4–73.4)	73.7%	<30, 30–49, >50	2328	patients undergoing PCI	UK	long-term
Gao [58]	2013	Prospective Observational	59.9 (11.1)	83.2%	<40, ≥40	4335	patients undergoing PCI	China	36 months
Halkin [54]	2005	Retrospective Observational	62 (53–71)	72.9%	<40, 40–50, 50–60, >60	1620	AMI	USA	1 year
Jackson [41]	2018	Retrospective Observational	68 (12)	77.0%	<30, 30–50, >50	260,726	patients who received PCI	UK	1 month
Keelan [11]	2003	Retrospective Observational		72.3%	≤40, 41–49, ≥50	1158	patients who underwent PCI	USA	in-hospital
Kwok [42]	2015	Retrospective Observational		73.5%	<30, 30–49, ≥50	246,840	patients who received PCI	UK	30 days
Levi [55]	2016	Retrospective Observational	72 (12)	73.0%	<30, 30–50, ≥50	974	patients who underwent an elective PCI	Israel	5 years
Mamas [14]	2014	Retrospective Observational	68.5 (68.3–68.6)	77.4%	<30, 30–50, >50	230,464	patients undergoing PCI for elective STEMI and non-STEMI	UK	5 years
Marsico [43]	2003	Retrospective Observational	67 (27–89)	79.2%	≤35, >35	2488	patients who underwent PTCA	Italy	in-hospital
Singh [44]	2007	Retrospective Observational	66.9 (12.1)	69.0%	<20, 20–39, 40–59, ≥60	7457	patients who underwent PCI	USA	in-hospital
van der Vleuten [33]	2008	secondary analysis of two randomized controlled trials	59.8 (12.0)	77.8%	<35, 35–55, >55	924	patients with STEMI treated with PCI	Israel	2.5 years
Wallace [45]	2009	Retrospective Observational	63.8 (11.7)	67.5%	<25, 26–35, 36–45, 46–55, >55	55,709	patients who underwent PCI	USA	in-hospital
Studies	33					969,868			

AMI, acute myocardial infarction; CTO, chronic total occlusion; LVEF, left ventricular ejection fraction; PCI, percutaneous coronary intervention; PTCA, Percutaneous Transluminal Coronary Angioplasty; STEMI, ST-elevation myocardial infarction.

### 3.3 Definition of LVEF Stratification

Normal LVEF is defined as LVEF ≥50% with and abnormal LVEF being defined as LVEF <50%. Abnormal LVEF is classified into moderately impaired LV function (LVEF 30–49%) and poor LV function (LVEF <30%). Because stratification according to LVEF slightly varied from study to study, the definition of stratification fluctuated + or –5% in our combined analysis.

### 3.4 All-Cause Mortality

The hazard ratios of all-cause mortality within 30 days (or in-hospital), in one year and over a period more than 1 year after PCI between patients with abnormal and normal LVEF were 2.96 [95% CI, 2.2, 3.98], 3.14 [95% CI, 1.64, 6.01] and 3.08 [95% CI, 2.6, 3.64]. The hazard ratios of all-cause mortality within 30 days (or in-hospital), in one year and over a period more than 1 year after PCI between patients with moderately impaired LV function and patients with normal LVEF were 2.32 [95% CI, 1.85, 2.91], 2.04 [95% CI, 1.37, 3.03], 1.93 [95% CI, 1.54, 2.44]. The hazard ratios of all-cause mortality within 30 days (or in-hospital), in one year and over a period more than 1 year after PCI between patients with poor LV function and patients with normal LVEF were 4.84 [95% CI, 3.83, 6.1], 4.48 [95% CI, 1.37, 14.68], 6.59 [95% CI, 4.23, 10.27] (Table 1, **Supplementary Figs. 1–9**). The above comparisons suggested that the poorer baseline LV function was a major source for all-cause PCI mortality.

### 3.5 Incidence of MACE

The odds ratios of MACE occurrence within 30 days (or in-hospital), in 1 year and over a period greater than 1 year after PCI between patients with abnormal and normal LVEF were 1.9 [95% CI, 1.65, 2.2], 1.71 [95% CI, 1.13, 2.59], and 1.37 [95% CI, 1.14, 1.65]. The odds ratios of MACE occurrence within 30 days (or in-hospital), in 1 year and over a long-term of period greater than 1 year after PCI between patients with moderately impaired LV function and patients with normal LVEF were 1.35 [95% CI, 1.27, 1.43], 1.194 [95% CI, 0.96, 1.48], and 1.15 [95% CI, 0.879, 1.52]. The odds ratios of MACE occurrence within 30 days (or in-hospital), in 1 year and over a period greater than 1 year after PCI between patients with poor LV function and patients with normal LVEF were 2.41 [95% CI, 2.04, 2.85], 1.47 [95% CI, 1.03, 2.081], and 2.31 [95% CI, 1.46, 3.66] (Table 2, **Supplementary Figs. 10–18**). The above comparisons suggest that the risk of MACE occurrence in 1 year or over a period greater than 1 year after PCI in patients with modestly impaired LV function was not different from that in patients with normal LVEF, but was greater in patients with poor baseline LV function than that in patients with normal LVEF.

**Table 2.** **Outcomes**.

Outcomes	Comparisons (stratified according to LVEF at baseline)	Follow-up	HR [95% CI]	*p*-value	I²	Studies	Samples
All-cause mortality	abnormal vs normal	30-day	2.96 [2.2, 3.98]	0.000	96.3%	13	813,975
All-cause mortality	abnormal vs normal	1-year	3.14 [1.64, 6.01]	0.000	99.7%	8	324,723
All-cause mortality	abnormal vs normal	long-term	3.08 [2.6, 3.64]	0.000	88.5%	7	237,097
All-cause mortality	moderate vs normal	30-day	2.32 [1.85, 2.91]	0.000	80.8%	9	807,277
All-cause mortality	moderate vs normal	1-year	2.04 [1.37, 3.03]	0.000	98.1%	7	320,026
All-cause mortality	moderate vs normal	long-term	1.93 [1.54, 2.44]	0.000	89.1%	5	235,665
All-cause mortality	poor vs normal	30-day	4.84 [3.83, 6.1]	0.000	77.8%	7	797,443
All-cause mortality	poor vs normal	1-year	4.48 [1.37, 14.68]	0.000	99.8%	5	317,248
All-cause mortality	poor vs normal	long-term	6.59 [4.23, 10.27]	0.000	96.7%	5	235,665
MACE	abnormal vs normal	30-day	1.9 [1.65, 2.2]	0.000	76.3%	7	521,584
MACE	abnormal vs normal	1-year	1.71 [1.13, 2.59]	0.011	61.2%	3	6477
MACE	abnormal vs normal	long-term	1.37 [1.14, 1.65]	0.001	0.0%	4	14,334
MACE	moderate vs normal	30-day	1.35 [1.27, 1.43]	0.000	2.3%	4	515,998
***MACE***	***moderate vs normal***	***1-year***	***1.19 [0.96, 1.48]***	***0.107***	***0.0%***	***2***	***6176***
***MACE***	***moderate vs normal***	***long-term***	***1.15 [0.87, 1.52]***	***0.329***	***0.0%***	***3***	***3844***
MACE	poor vs normal	30-day	2.41 [2.04, 2.85]	0.000	61.3%	4	515,998
MACE	poor vs normal	1-year	1.46 [1.03, 2.08]	0.036	0.0%	2	6176
MACE	poor vs normal	long-term	2.31 [1.46, 3.66]	0.000	0.0%	2	1814
Cardiac death	<40 vs >40	30-day	7.54 [2.7, 21.06]	0.000	56.1%	2	6765
Cardiac death	<40 vs >40	1-year	4.51 [1.96, 10.38]	0.000	0.0%	2	8457
Cardiac death	<40 vs >40	long-term	6.51 [4.25, 9.97]	0.000	51.2%	3	10,887
CTO-Death	abnormal vs normal	all	3.3 [2.53, 4.29]	0.000	0.0%	2	2841
CTO-MACE	abnormal vs normal	all	1.6 [1.34, 1.9]	0.000	0.0%	3	2916
STEMI-Death	abnormal vs normal	30-day	4.36 [1.52, 12.5]	0.000	96.9%	4	264,475
STEMI-Death	abnormal vs normal	1-year	5.22 [3.87, 7.04]	0.000	92.8%	3	233,818
STEMI-Death	abnormal vs normal	long-term	3.83 [3.35, 4.37]	0.000	82.7%	3	233,818
STEMI-MACE	abnormal vs normal	30-day	3.78 [2.54, 5.64]	0.000	0.0%	2	2679
HF-Death	HFrEF vs HFpEF	30-day	1.36 [1.15, 1.6]	0.000	0.0%	3	34,097

CTO, chronic total occlusion; HF, heart failure; HFrEF, HF with reduced ejection fraction; HFpEF, HF with preserved ejection fraction; HR, Hazard ratio; LVEF, left ventricular ejection fraction; MACE, major adverse cardiac events; STEMI, ST-elevation myocardial infarction. Bold italics mean no statistical significance.

### 3.6 Cardiovascular Mortality

Due to the paucity of study data, in this study the outcomes were pooled based on a cutoff value of 40% for the baseline LVEF. The hazard ratios of cardiovascular mortality within 30 days, in 1 year and over a period greater than 1 year after PCI between patients with baseline LVEF <40% and patients with baseline LVEF ≥40% were 7.54 [95% CI, 2.7, 21.06], 4.507 [95% CI, 1.96, 10.38], 6.51 [95% CI, 4.25, 9.97]. The results indicate that patients with baseline LVEF <40% have a higher risk of short- and long-term cardiovascular mortality after PCI (Table 2, **Supplementary Figs. 19–21**).

### 3.7 Subgroup Analysis

Among patients undergoing elective CTO PCI, patients with abnormal LVEF had significantly higher all-cause mortality than that of patients with normal LVEF, HR = 3.30 [95% CI, 2.53, 4.29], and the incidence of MACE was significantly increased, OR = 1.60 [95% CI, 1.34, 1.90]. Among patients undergoing STEMI PCI, patients with abnormal LVEF had significantly higher 30-day, 1-year, and long-term all-cause mortality compared to patients with normal LVEF, with HR = 4.36 [95% CI, 1.52, 12.5], 5.22 [95% CI, 3.87, 7.04], and 3.83 [95% CI, 3.35, 4.37]. Compared with HFpEF, patients with HFrEF undergoing PCI were significantly noted to have an increased all-cause mortality, HR = 1.36 [95% CI, 1.14, 1.60] (Table 2, **Supplementary Figs. 22–28**).

### 3.8 Publication Bias

Publication bias was assessed using funnel plots and asymmetry of the funnel plot was evaluated with the Egger regression test for implementation strategies with at least 10 studies. We found publication bias in the comparison of 30-day all-cause mortality in patients with abnormal versus normal LVEF (**Supplementary Fig. 29**).

## 4. Discussion

A total of 969,868 participants in 33 studies [10, 11, 13, 14, 30, 31, 32, 33, 34, 35, 36, 37, 38, 39, 40, 41, 42, 43, 44, 45, 46, 47, 48, 49, 50, 51, 52, 53, 54, 55, 56, 57, 58] at different stratification of baseline LVEF were included in this study and their PCI outcomes were stratified for analysis. This study found that lower baseline LVEF was associated with higher risk of all-cause mortality after PCI. Patients with a moderate level of LVEF had a 2.32-fold increased risk of all-cause mortality within 30 days and a 1.93-fold increased risk of all-cause mortality over 1 year compared with patients with a normal LVEF. Compared with patients with normal LVEF, the HR of 30-day all-cause mortality was 4.84 and the HR of over 1-year all-cause mortality was 6.59 in patients with poor LVEF. We also investigated cardiovascular mortality in patients with LVEF below 40%. Our study demonstrated that patients with LVEF below 40% had 7.54 times higher 30-day cardiovascular mortality and 6.54 times higher cardiovascular mortality over 1 year compared with patients with LVEF above 40%. This data supports that reduced LVEF is an important contributing factor to the prognosis of all-cause mortality, especially to cardiovascular death after PCI.

Studies have suggested that our findings may be related to the following reasons: (1) patients with reduced LV function are mostly elderly diabetic patients with a history of acute myocardial infarction and a higher possibility of cardiogenic shock; (2) with the decrease of LV function, the shear forces in the stented segment decreases, increasing the possibility of thrombosis [59, 60]; (3) with the decline of cardiac function, the incidence of renal insufficiency, which is a known risk factor for stent thrombosis, increases. In view of this, adequate preoperative evaluation of LVEF and the pursuit of optimal revascularization may be of great significance for the outcomes of these patients. Some guidelines recommend CABG as a revascularization strategy for patients with poor ejection fraction. The European Society Of Cardiology Guidelines indicate that CABG is superior to PCI, whereas the US guidelines only recommend CABG and have no comment on PCI [61, 62]. CABG is more likely to achieve complete revascularization than PCI [63]. Full revascularization can more effectively reduce the burden of myocardial ischemia, thereby reducing the risk of both sudden death and cardiac death. Moreover, CABG is better for blood supply in the distal vascular bed with full revascularization achieved after CABG resulting in better outcomes to patients [64]. However, PCI is still widely used due to its operational ease and patients’ own choice. Especially in the context of ACS, evaluation of LVEF before PCI becomes even more important when clinicians are challenged to complete surgery within 72 [18] or 48 hours [19]. Understanding LV dysfunction can provide insight into the possible complexity of the intended PCI, thus providing a basis for preoperative preparation and medical optimization of patients. Meanwhile, this ensures that all team members understand the high-risk nature of the case before starting the procedure, and deploy percutaneous LV assist device in advance, such as axial flow pumpor intra-aortic balloon counter-pulsation. Moreover, identifying high-risk patients facilitates postoperative care, understanding postoperative changes in LVEF, and increases the use of more appropriate postinfarction therapies, such as optimal doses of angiotensin receptor blockers, aldosterone antagonists, or angiotensin converting enzyme inhibitors [65, 66]. The ESC Clinical Practice Guidelines advise that all patients with STEMI undergo a systematic echocardiographic assessment to assess LV function before discharge from the hospital. For patients with LVEF ≤40%, the guidelines call for an early re-evaluation within 6–12 weeks after leaving the hospital to assess the need for device-based interventions [67, 68]. Data support that the assessment of LVEF before PCI (including ACS patients) is extremely significant for the success of PCI and the improvement of patients’ postoperative survival.

Our subgroup analysis of CTO PCI patients suggests that CTO patients with abnormal LVEF have a 3.3-fold increased risk of mortality and a 1.6-fold increased risk of MACE compared with CTO patients with normal LVEF. PCI may provide significant clinical benefit for CTO [69]. Although the applicability of CTO PCI to symptomatic patients has been generally accepted by guidelines and consensus [70], CTO PCI has another important potential benefit, that is improved LV function. Preoperative assessment of LVEF is necessary to assess the improvement in LV function. According to Galassi *et al*. [35] and Tajstra *et al*. [71], a higher prevalence of peripheral vascular disease, chronic kidney disease, and diabetes mellitus in patients with CTO and low LVEF significantly increases the surgical risk. Preoperative LVEF assessment is critical to identify high-risk patients who are to undergo CTO PCI. CTO PCI is a relatively complex procedure, and blocking side branches during CTO PCI is associated with a high risk of coronary perforation and perforation tamponade [72] as well as periprocedural myocardial infarction [73, 74]. The use of the antegrade crossing techniques in CTO recanalization may be preferable because the retrograde crossing techniques have been associated with a high risk of procedural complications [74, 75] and surgical perioperative myocardial infarction. However, preservation of bifurcations and recanalization of complex CTOs often require retrograde techniques [76, 77]. CTO PCI relies heavily on operator experience, so preoperative LVEF evaluation is necessary to fully understand the patient’s condition and the difficulty of operation.

Our subgroup analysis of HF patients showed that the 30-day mortality in HFrEF patients was 1.36 times higher than that in HFpEF patients. Although the data volume is small, it can still be seen that HFrEF patients have a poor prognosis. Currently, there are no clear guidelines for the role of PCI in the treatment of HFrEF. Therefore, for HF patients, pre-PCI LVEF assessment is needed to identify HFrEF patients and select appropriate treatment strategies.

Our findings have some limitations. The stratification criteria of LVEF were not completely consistent across the included studies, and relatively broad criteria were used to classify LVEF into normal, moderate and poor levels, which may account for the higher heterogeneity (Table 2). The time span of our included studies was 20 years and during this time many advances have been made in PCI technology, which may also be one of the reasons for the high heterogeneity among studies in different years. Therefore, the effect sizes in the meta-analysis of all-cause mortality should be interpreted with caution. However, a random effects model has been used to minimize the bias associated with high heterogeneity.

## 5. Conclusions

Our study suggests that a moderate or severe reduction in patients’ LVEF may have a serious impact on PCI prognosis. Therefore, we strongly advocate for adequate assessment of LVEF before PCI (regardless of ACS) in order to choose the optimal revascularization and postoperative treatment resulting in reduced short- and long-term mortality.

## Abbreviations

ACS, acute coronary syndrome(s); BMS, bare-metal stent; CABG, coronary artery bypass grafting; CHF, Congestive heart failure; CTO, chronic total occlusion; DES, drug eluting stent; HF, heart failure; HFpEF, HF with preserved ejection fraction; HFrEF, HF with reduced ejection fraction; HR, Hazard ratio; LV, left ventricular; LVEF, left ventricular ejection fraction; MACE, major adverse cardiac events; NOS, Newcastle-Ottawa Quality Assessment Scale; OR, odds ratio; PCI, percutaneous coronary intervention; STEMI, ST-elevation myocardial.

## Supplementary Material

Supplementary material associated with this article can be found, in the online version, at https://doi.org/10.31083/j.rcm2403080.
