# Supplementary material for: Prognostic Value of Preoperative Assessment of Left Ventricular Function in Patients Undergoing Percutaneous Coronary Intervention
Source: Rev Cardiovasc Med. 2023 Mar 6;24(3):80. doi: 10.31083/j.rcm2403080 (PMC11264021; doi:10.31083/j.rcm2403080)
Supplement: Supplementary file 1 [file 2153-8174-24-3-080-s1.zip › 2153-8174-24-3-080-s1/Supplementary Material.docx]

**Supplementary Material**

**Supplementary Method M1** Search term in Scopus

( TITLE-ABS-KEY ( pci ) OR TITLE-ABS-KEY ( coronary AND intervention )) AND (TITLE-ABS-KEY ( lvef ) OR TITLE-ABS-KEY ( ejection AND fraction ) OR TITLE-ABS-KEY ( lv and function) OR TITLE-ABS-KEY ( left and ventricular and function) OR TITLE-ABS-KEY ( HFrEF) OR TITLE-ABS-KEY (HFpEF)) AND (TITLE-ABS-KEY ( prognosis ) OR TITLE-ABS-KEY ( Prognostic) OR TITLE-ABS-KEY ( outcomes) OR TITLE-ABS-KEY ( events) OR TITLE-ABS-KEY ( death) OR TITLE-ABS-KEY ( hospitalization) OR TITLE-ABS-KEY ( Survival) OR TITLE-ABS-KEY ( Mortality) OR TITLE-ABS-KEY ( Readmission))

**Supplementary Table 1** The Newcastle-Ottawa Quality Assessment Scale for assessing the quality of studies

| Study | Year | Selection | Comparability | Outcome | Score |
| --- | --- | --- | --- | --- | --- |
| Alidoosti [1] | 2008 | ★★★★ | ★★ | ★★★ | 9 |
| Banga [2] | 2019 | ★★★ | ★ | ★★ | 6 |
| Daneault [3] | 2013 | ★★★ | ★★ | ★★★ | 8 |
| Doshi [4] | 2019 | ★★★★ | ★★ | ★★★ | 9 |
| El Awady [5] | 2020 | ★★★ | ★ | ★ | 5 |
| Galassi [6] | 2017 | ★★★ | ★★ | ★★ | 7 |
| Holper [7] | 2006 | ★★★ | ★★ | ★★★ | 8 |
| Jiang [8] | 2017 | ★★★★ | ★ | ★★★ | 8 |
| Jiang [9] | 2019 | ★★★ | ★★ | ★★ | 7 |
| Marui [10] | 2014 | ★★★ | ★ | ★★★ | 7 |
| Sardi [11] | 2012 | ★★★★ | ★ | ★★ | 7 |
| Shiga [12] | 2009 | ★★★★ | ★ | ★★★ | 8 |
| Son [13] | 2016 | ★★★ | ★★ | ★★★ | 8 |
| Sutton [14] | 2016 | ★★★★ | ★★ | ★★★ | 9 |
| Toma [15] | 2017 | ★★★★ | ★★ | ★★★ | 9 |
| Vakili [16] | 2014 | ★★★ | ★ | ★ | 5 |
| Wang [17] | 2017 | ★★★ | ★★ | ★★ | 7 |
| Ye [18] | 2018 | ★★★ | ★★ | ★★ | 7 |
| Zhong [19] | 2020 | ★★★ | ★ | ★★★ | 7 |
| Alaswad [20] | 2018 | ★★★ | ★ | ★★ | 6 |
| Biondi-Zoccai [21] | 2011 | ★★★ | ★ | ★★★ | 7 |
| De Silva [22] | 2012 | ★★★★ | ★★ | ★★★ | 9 |
| Gao [23] | 2013 | ★★★★ | ★ | ★★★ | 8 |
| Halkin [24] | 2005 | ★★★ | ★ | ★★★ | 7 |
| Jackson [25] | 2018 | ★★★★ | ★★ | ★★ | 8 |
| Keelan [26] | 2003 | ★★★ | ★★ | ★★ | 7 |
| Kwok [27] | 2015 | ★★★★ | ★★ | ★★★ | 9 |
| Levi [28] | 2016 | ★★★ | ★ | ★★★ | 7 |
| Mamas [29] | 2014 | ★★★★ | ★★ | ★★★ | 9 |
| Marsico [30] | 2003 | ★★★ | ★ | ★★ | 6 |
| Singh [31] | 2007 | ★★★★ | ★ | ★★★ | 8 |
| van der Vleuten [32] | 2008 | ★★★★ | ★★ | ★★★ | 9 |
| Wallace [33] | 2009 | ★★★★ | ★★ | ★★★ | 9 |

**Supplementary Fig. 1** Forest plot of hazard ratio of 30-day all-cause mortality among patients undergoing percutaneous coronary intervention (PCI), left ventricular ejection fraction (LVEF) abnormal vs normal


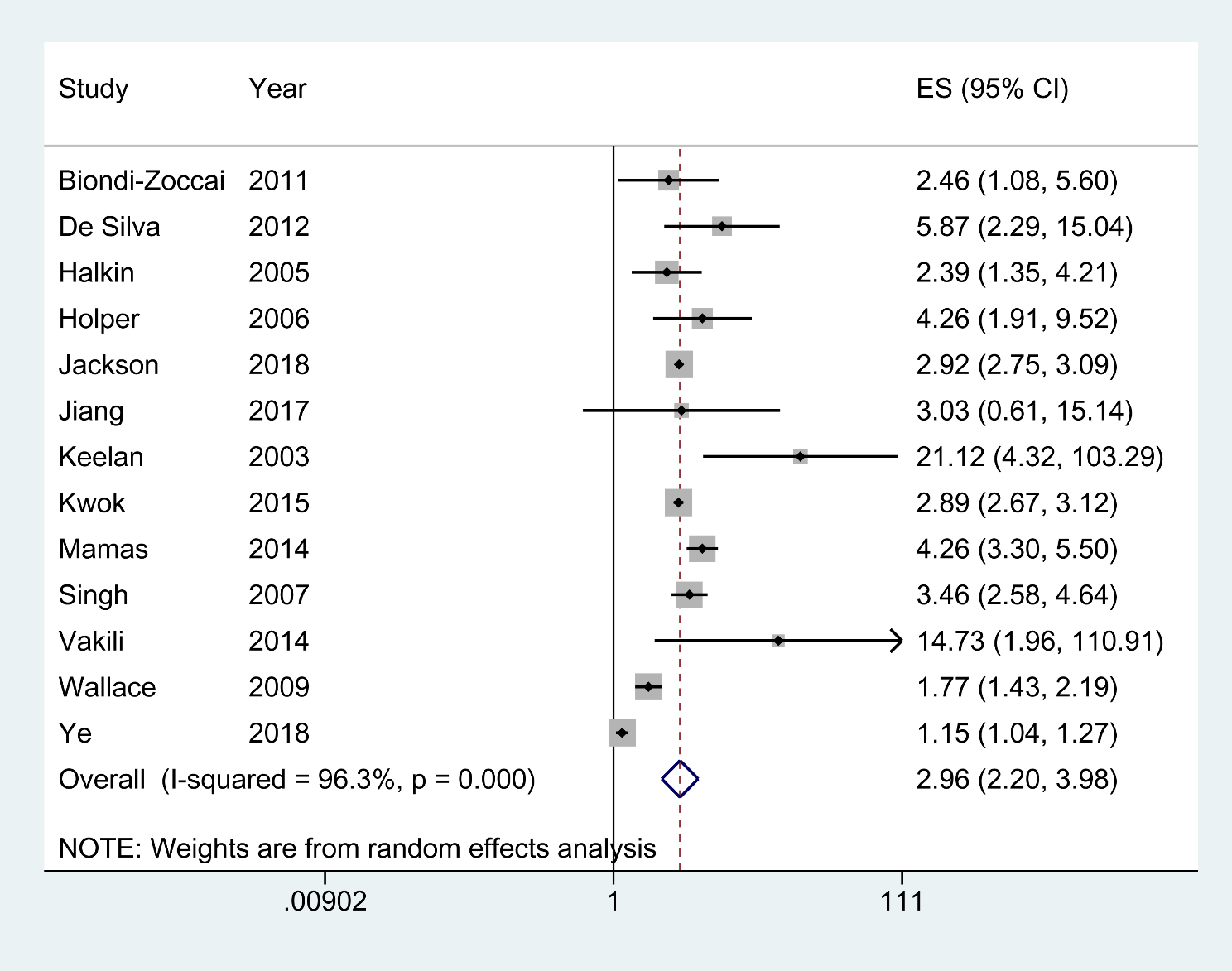


**Supplementary Fig. 2** Forest plot of hazard ratio of 1-year all-cause mortality among patients undergoing percutaneous coronary intervention (PCI), left ventricular ejection fraction (LVEF) abnormal vs normal


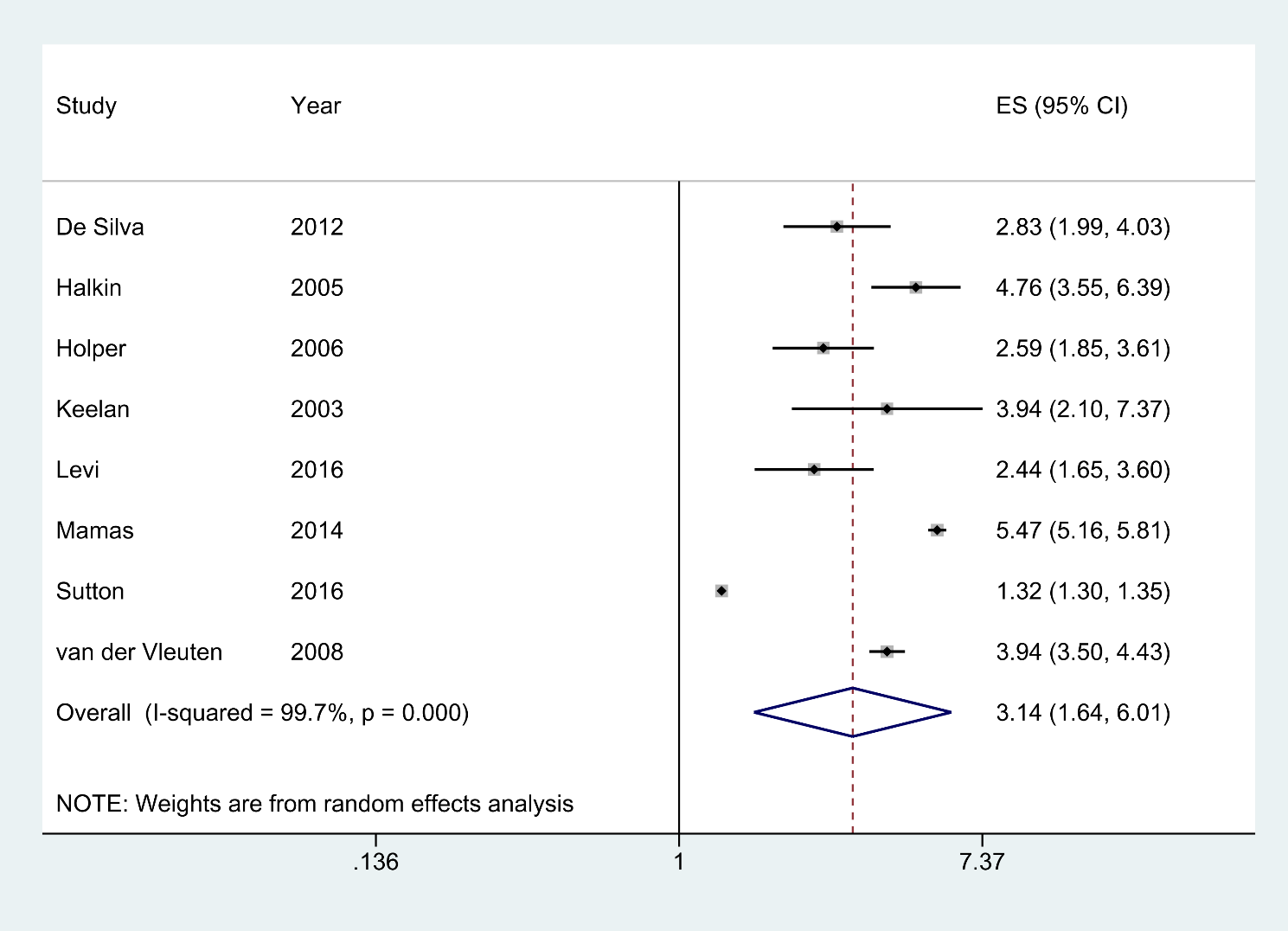


**Supplementary Fig. 3** Forest plot of hazard ratio of long-term all-cause mortality among patients undergoing percutaneous coronary intervention (PCI), left ventricular ejection fraction (LVEF) abnormal vs normal


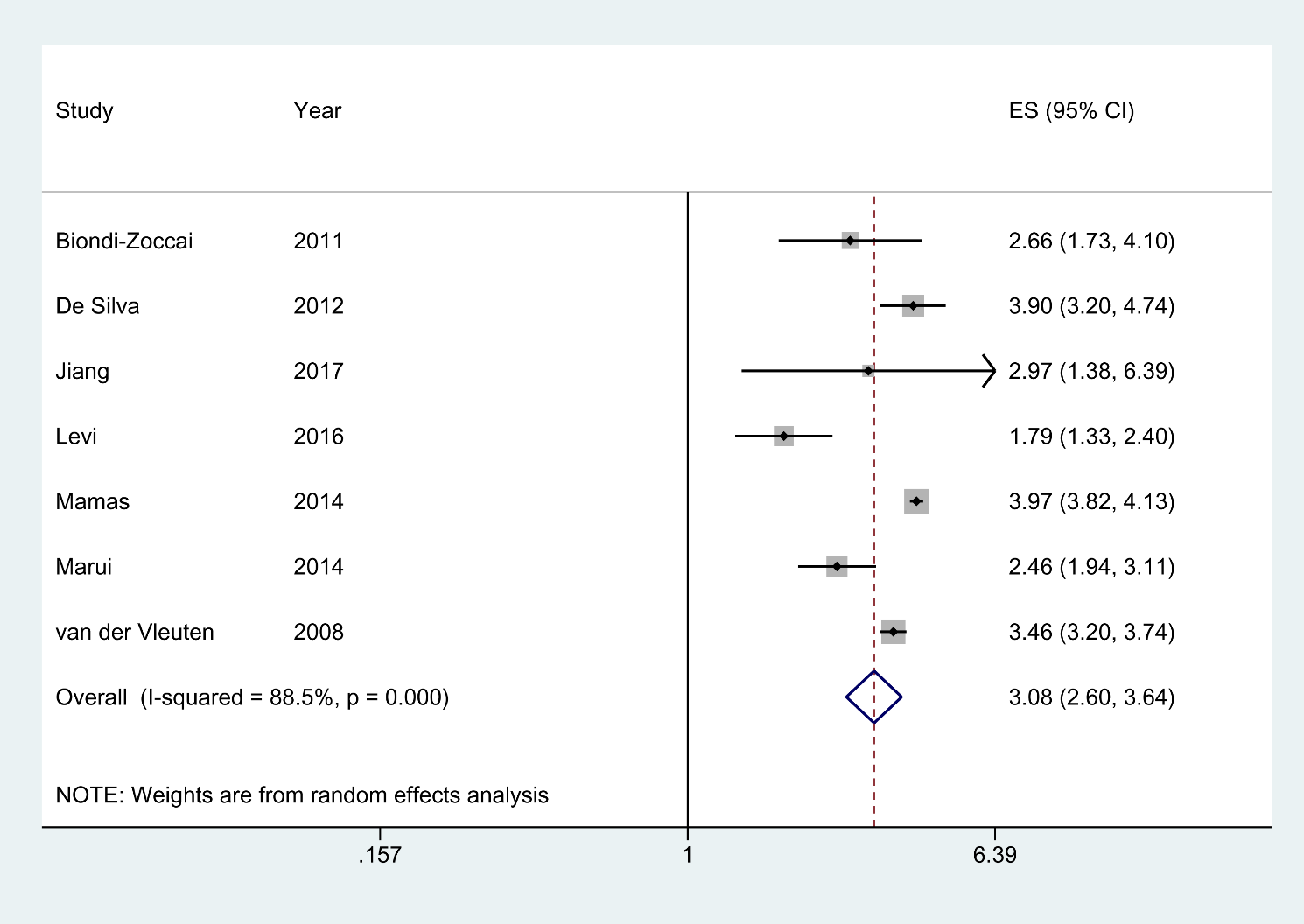


**Supplementary Fig. 4** Forest plot of hazard ratio of 30-day all-cause mortality among patients undergoing percutaneous coronary intervention (PCI), left ventricular ejection fraction (LVEF) moderate vs normal


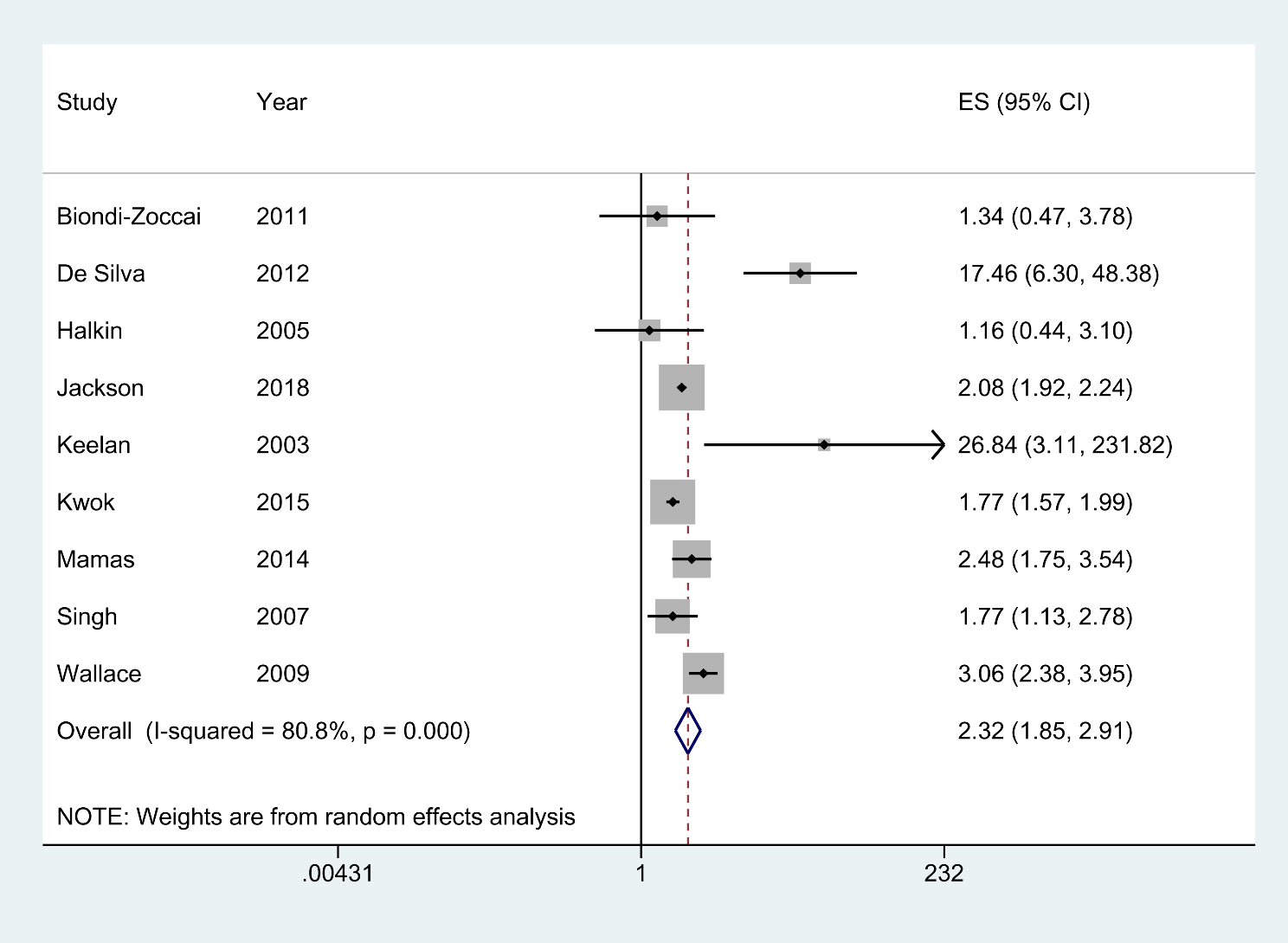


**Supplementary Fig. 5** Forest plot of hazard ratio of 1-year all-cause mortality among patients undergoing percutaneous coronary intervention (PCI), left ventricular ejection fraction (LVEF) moderate vs normal


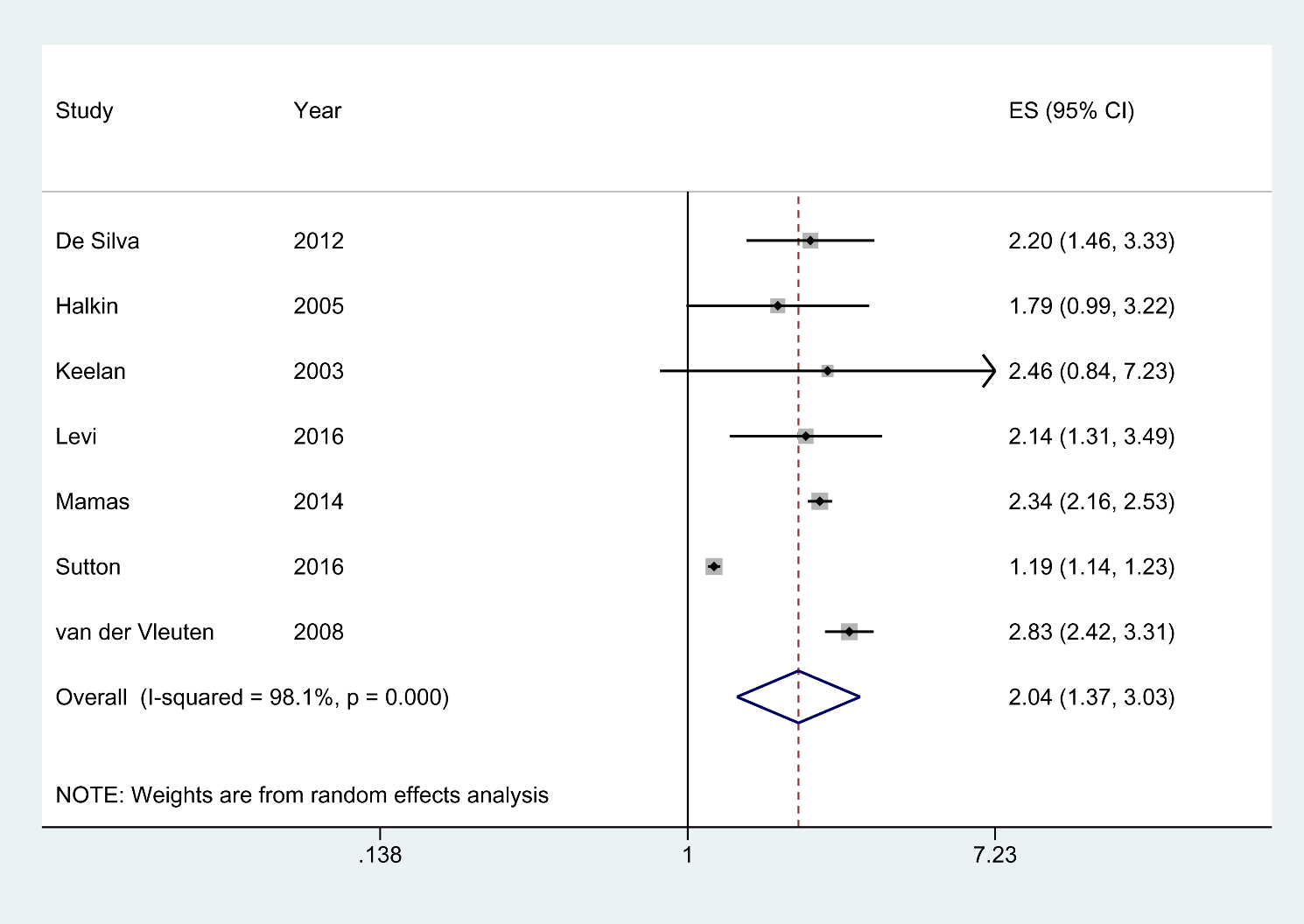


**Supplementary Fig. 6** Forest plot of hazard ratio of long-term all-cause mortality among patients undergoing percutaneous coronary intervention (PCI), left ventricular ejection fraction (LVEF) moderate vs normal


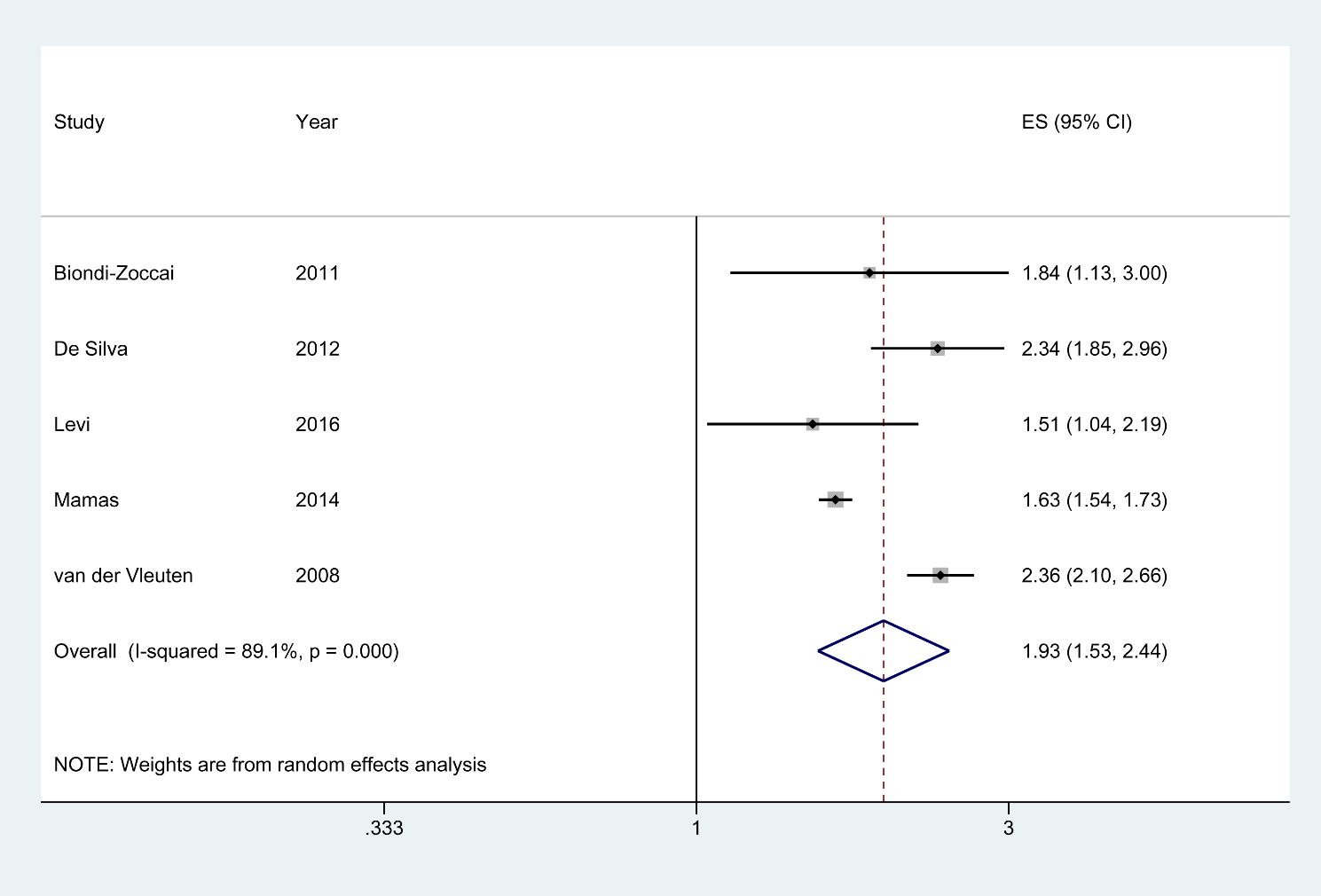


**Supplementary Fig. 7** Forest plot of hazard ratio of 30-day all-cause mortality among patients undergoing percutaneous coronary intervention (PCI), left ventricular ejection fraction (LVEF) poor vs normal


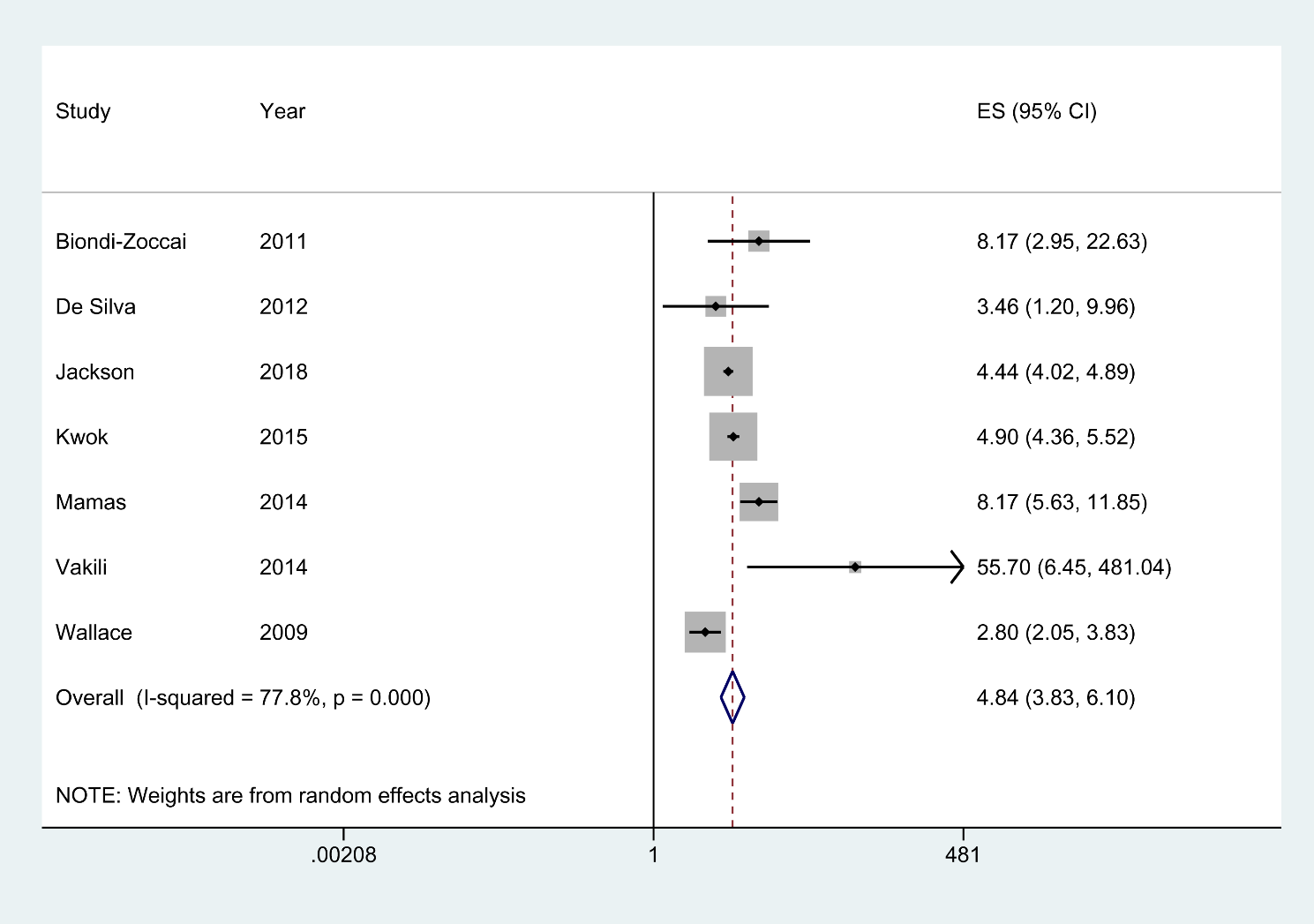


**Supplementary Fig. 8** Forest plot of hazard ratio of 1-year all-cause mortality among patients undergoing percutaneous coronary intervention (PCI), left ventricular ejection fraction (LVEF) poor vs normal


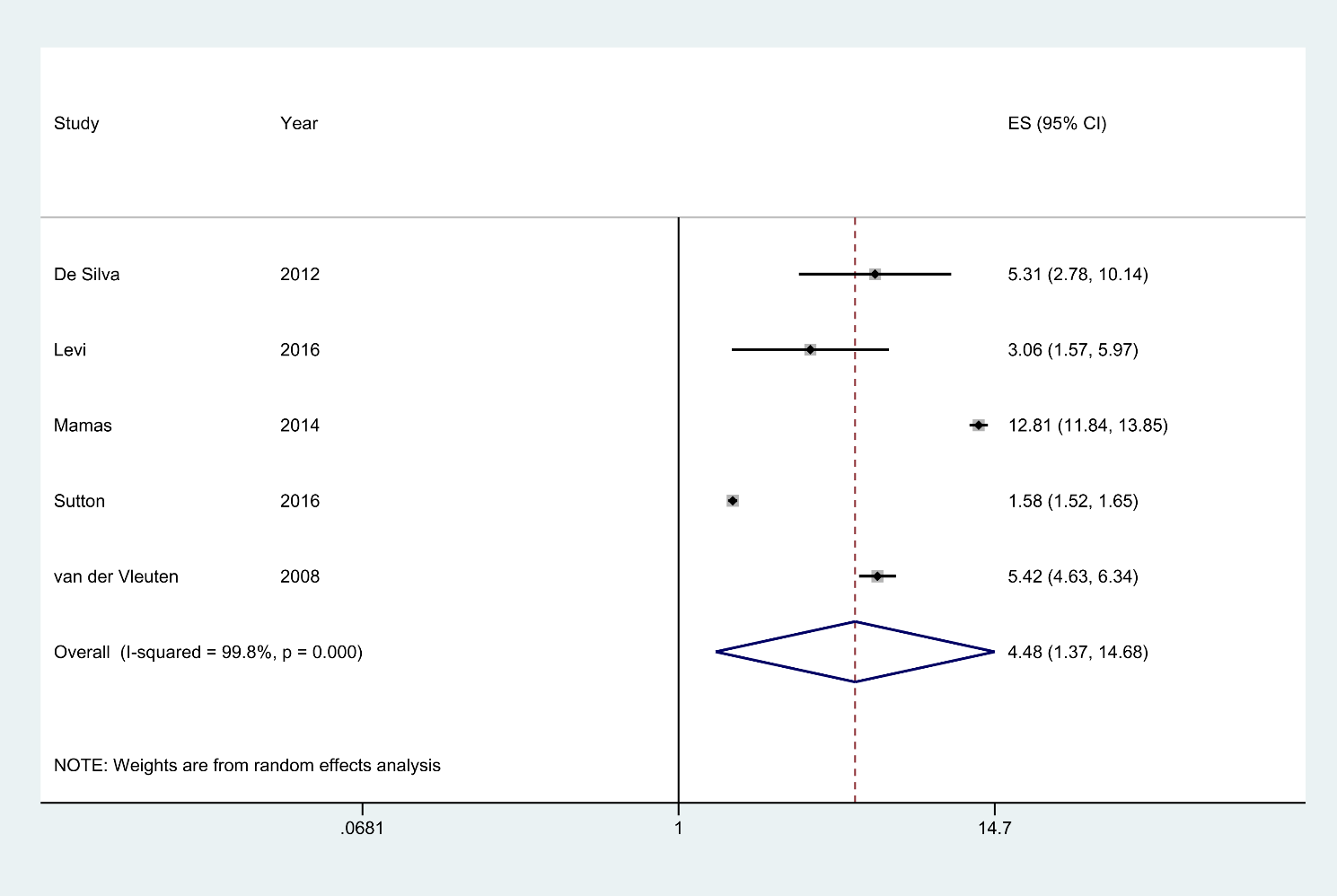


**Supplementary Fig. 9** Forest plot of hazard ratio of long-term all-cause mortality among patients undergoing percutaneous coronary intervention (PCI), left ventricular ejection fraction (LVEF) poor vs normal


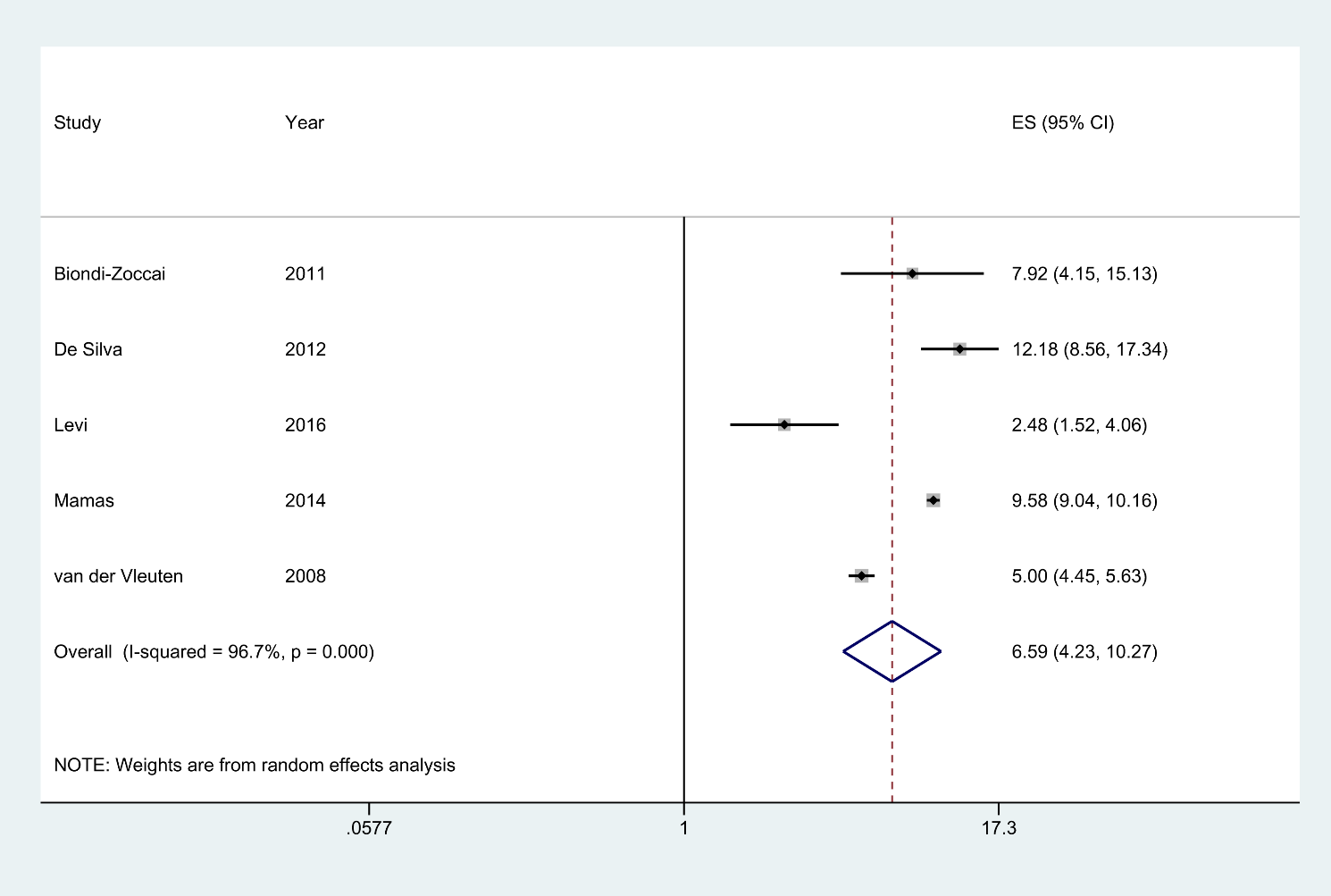


**Supplementary Fig. 10** Forest plot of odds ratio of 30-day major adverse cardiac events (MACE) among patients undergoing percutaneous coronary intervention (PCI), left ventricular ejection fraction (LVEF) abnormal vs normal


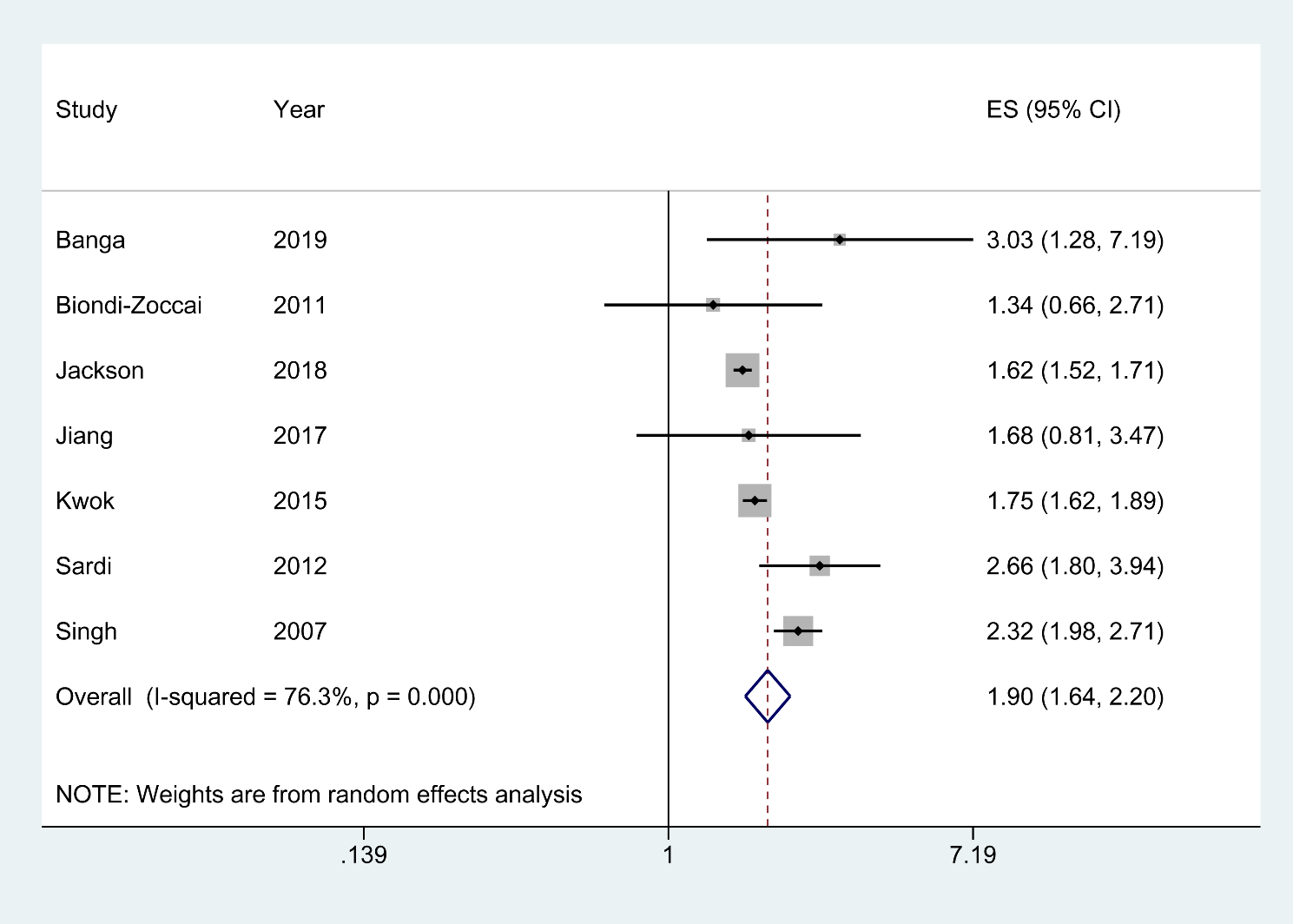


**Supplementary Fig. 11** Forest plot of odds ratio of 1-year major adverse cardiac events (MACE) among patients undergoing percutaneous coronary intervention (PCI), left ventricular ejection fraction (LVEF) abnormal vs normal


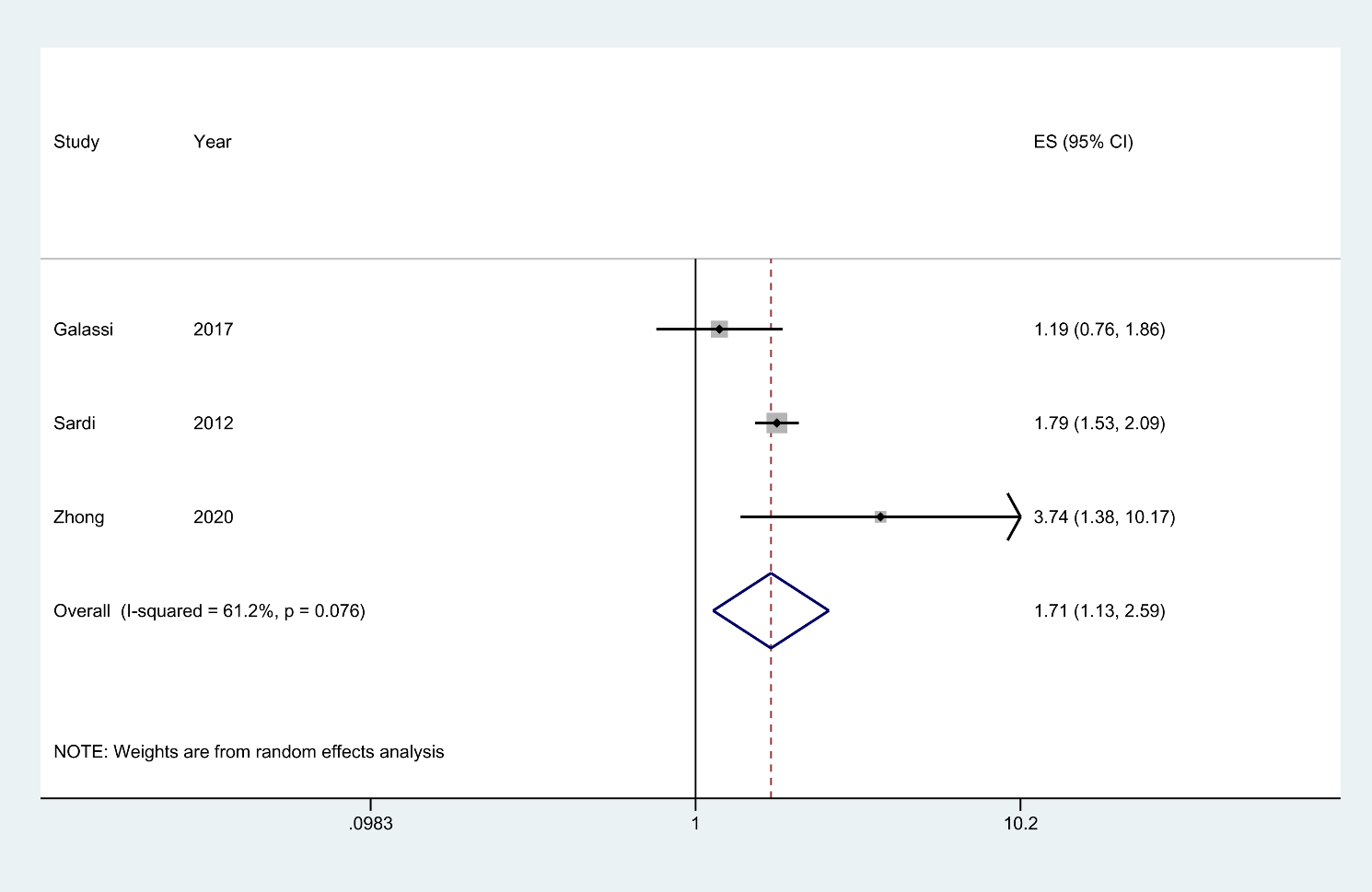


**Supplementary Fig. 12** Forest plot of odds ratio of long-term major adverse cardiac events (MACE) among patients undergoing percutaneous coronary intervention (PCI), left ventricular ejection fraction (LVEF) abnormal vs normal


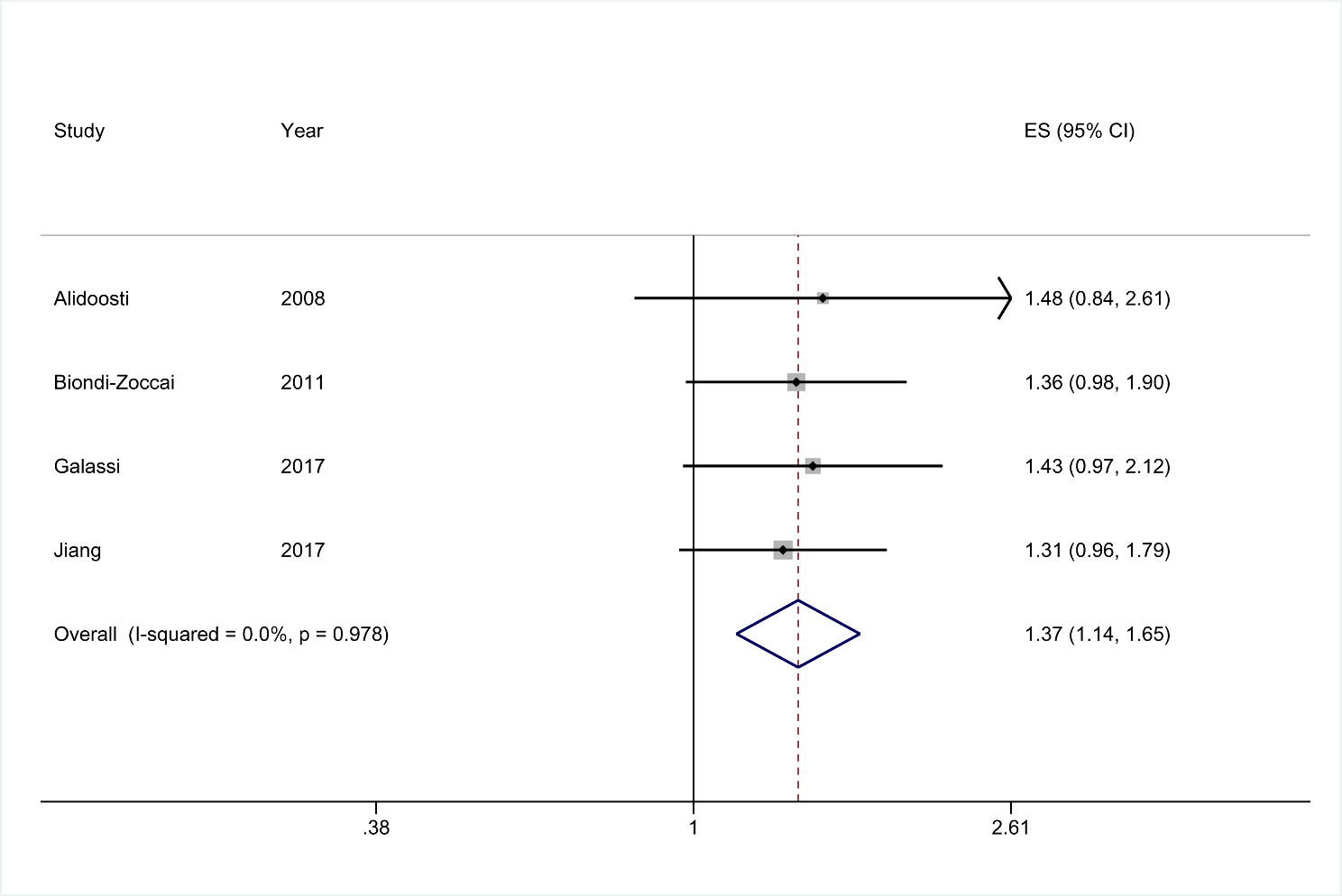


**Supplementary Fig. 13** Forest plot of odds ratio of 30-day major adverse cardiac events (MACE) among patients undergoing percutaneous coronary intervention (PCI), left ventricular ejection fraction (LVEF) moderate vs normal


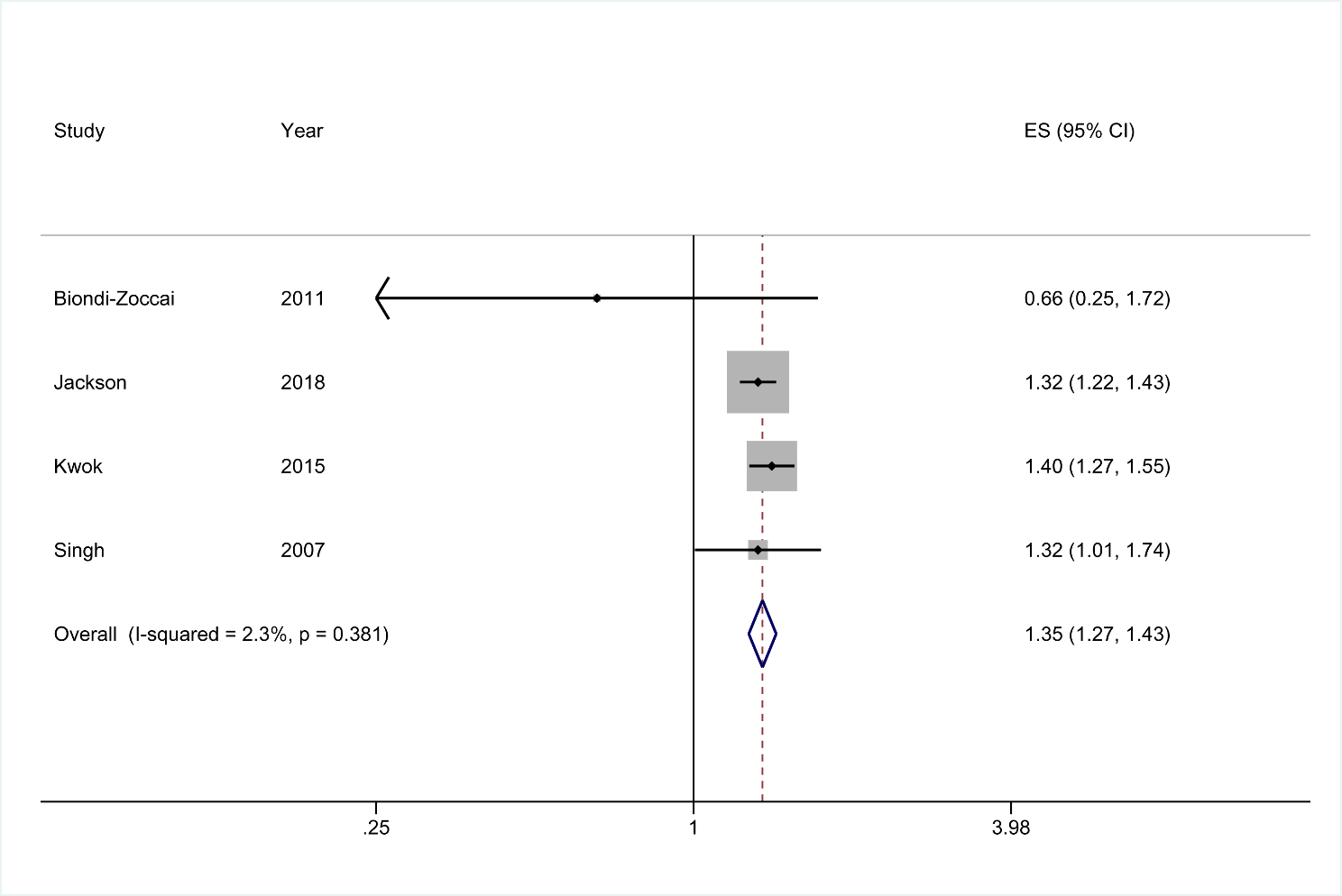


**Supplementary Fig. 14** Forest plot of odds ratio of 1-year major adverse cardiac events (MACE) among patients undergoing percutaneous coronary intervention (PCI), left ventricular ejection fraction (LVEF) moderate vs normal


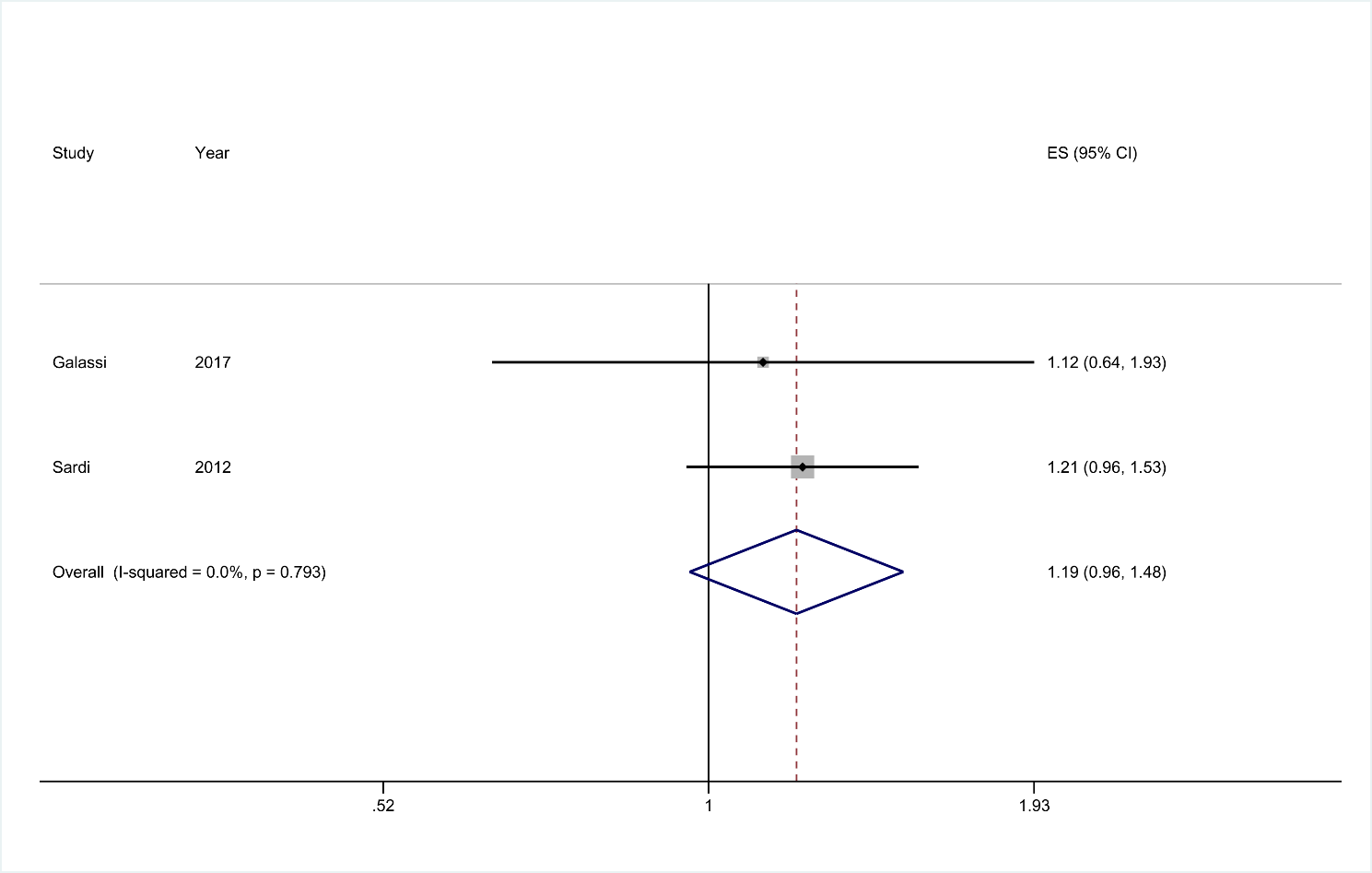


**Supplementary Fig. 15** Forest plot of odds ratio of long-term major adverse cardiac events (MACE) among patients undergoing percutaneous coronary intervention (PCI), left ventricular ejection fraction (LVEF) moderate vs normal


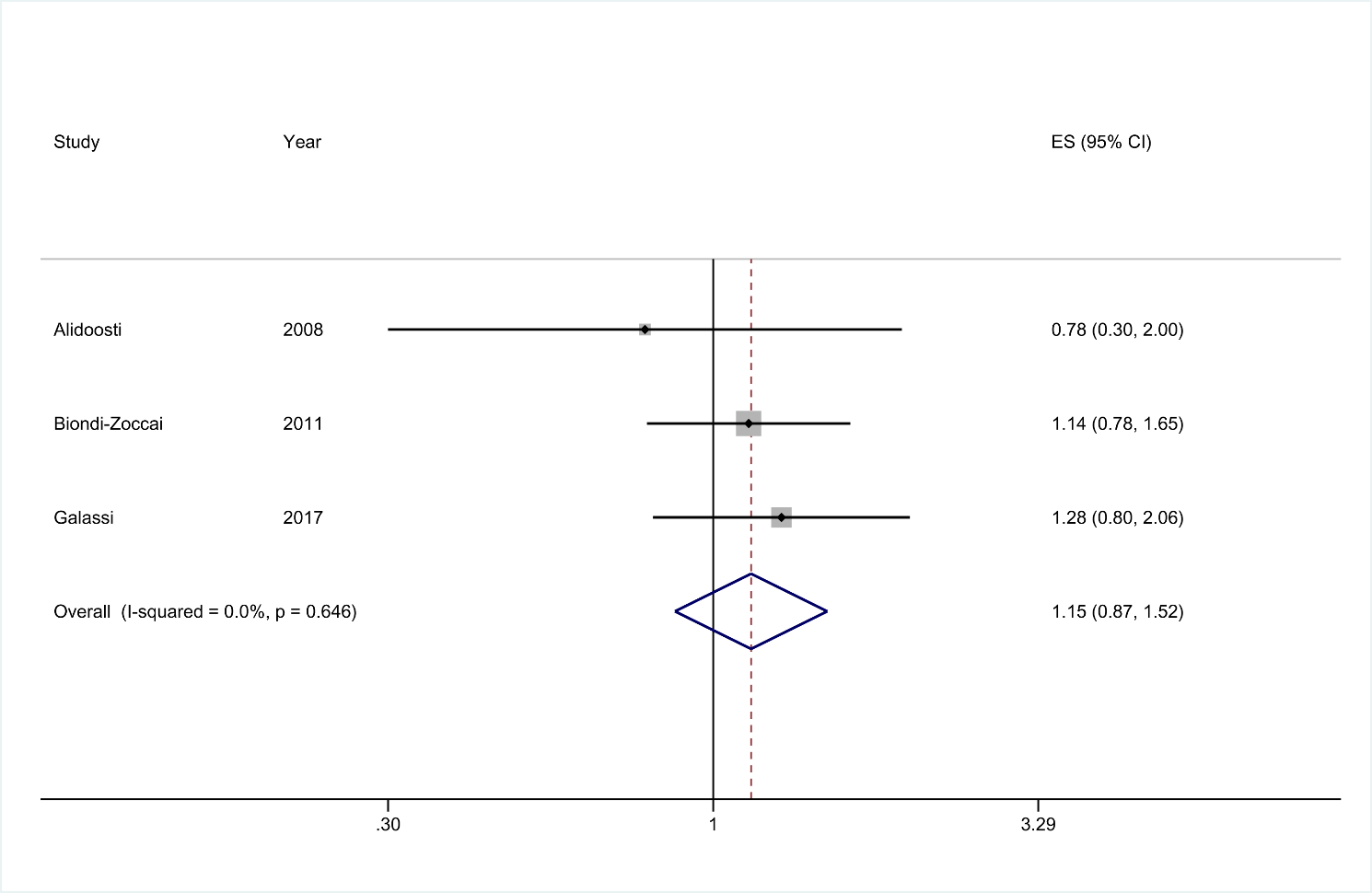


**Supplementary Fig. 16** Forest plot of odds ratio of 30-day major adverse cardiac events (MACE) among patients undergoing percutaneous coronary intervention (PCI), left ventricular ejection fraction (LVEF) poor vs normal


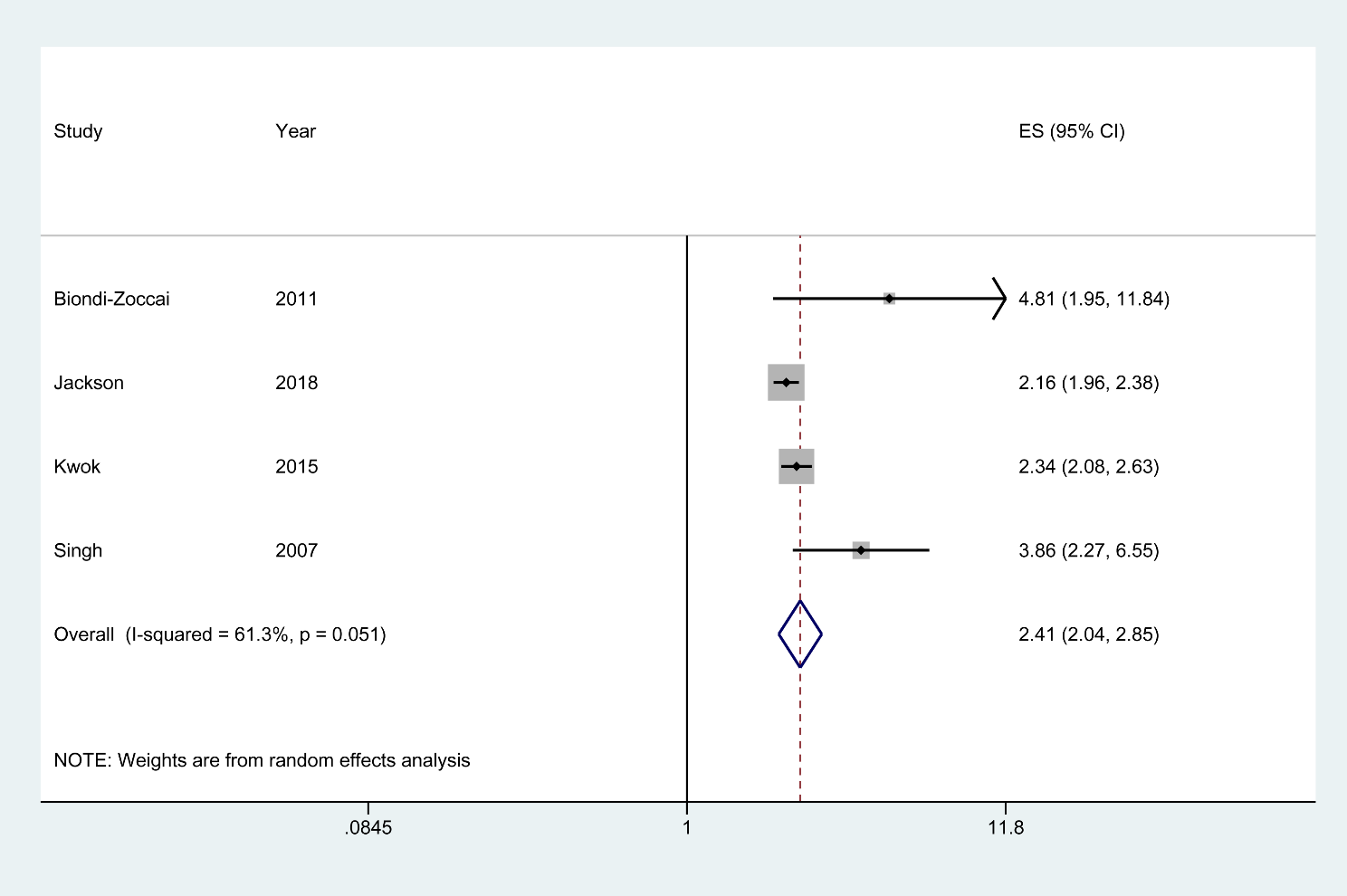


**Supplementary Fig. 17** Forest plot of odds ratio of 1-year major adverse cardiac events (MACE) among patients undergoing percutaneous coronary intervention (PCI), left ventricular ejection fraction (LVEF) poor vs normal


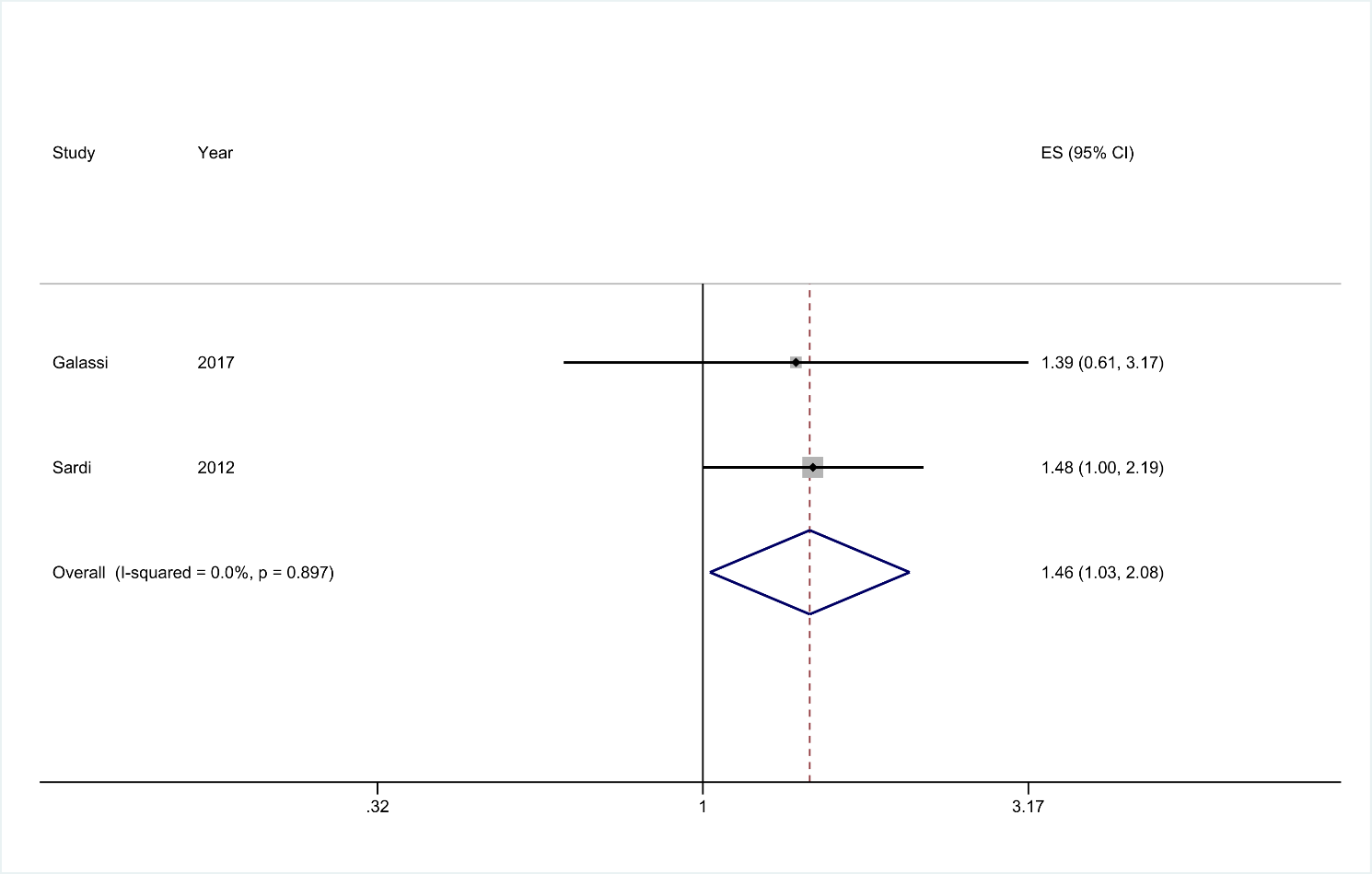


**Supplementary Fig. 18** Forest plot of odds ratio of long-term major adverse cardiac events (MACE) among patients undergoing percutaneous coronary intervention (PCI), left ventricular ejection fraction (LVEF) poor vs normal


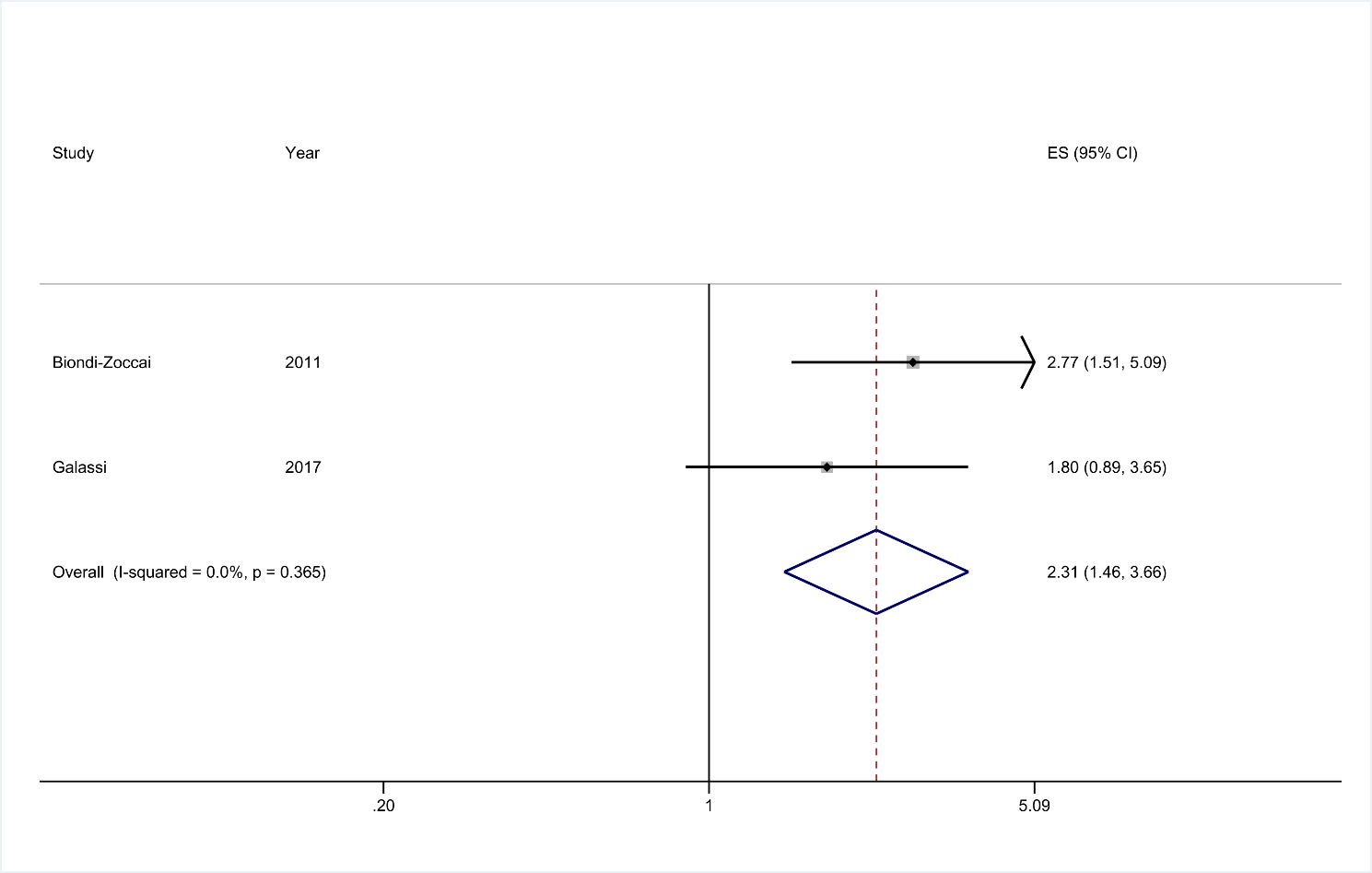


**Supplementary Fig. 19** Forest plot of hazard ratio of 30-day cardiac mortality among patients undergoing percutaneous coronary intervention (PCI), left ventricular ejection fraction (LVEF) under 40% vs above 40%


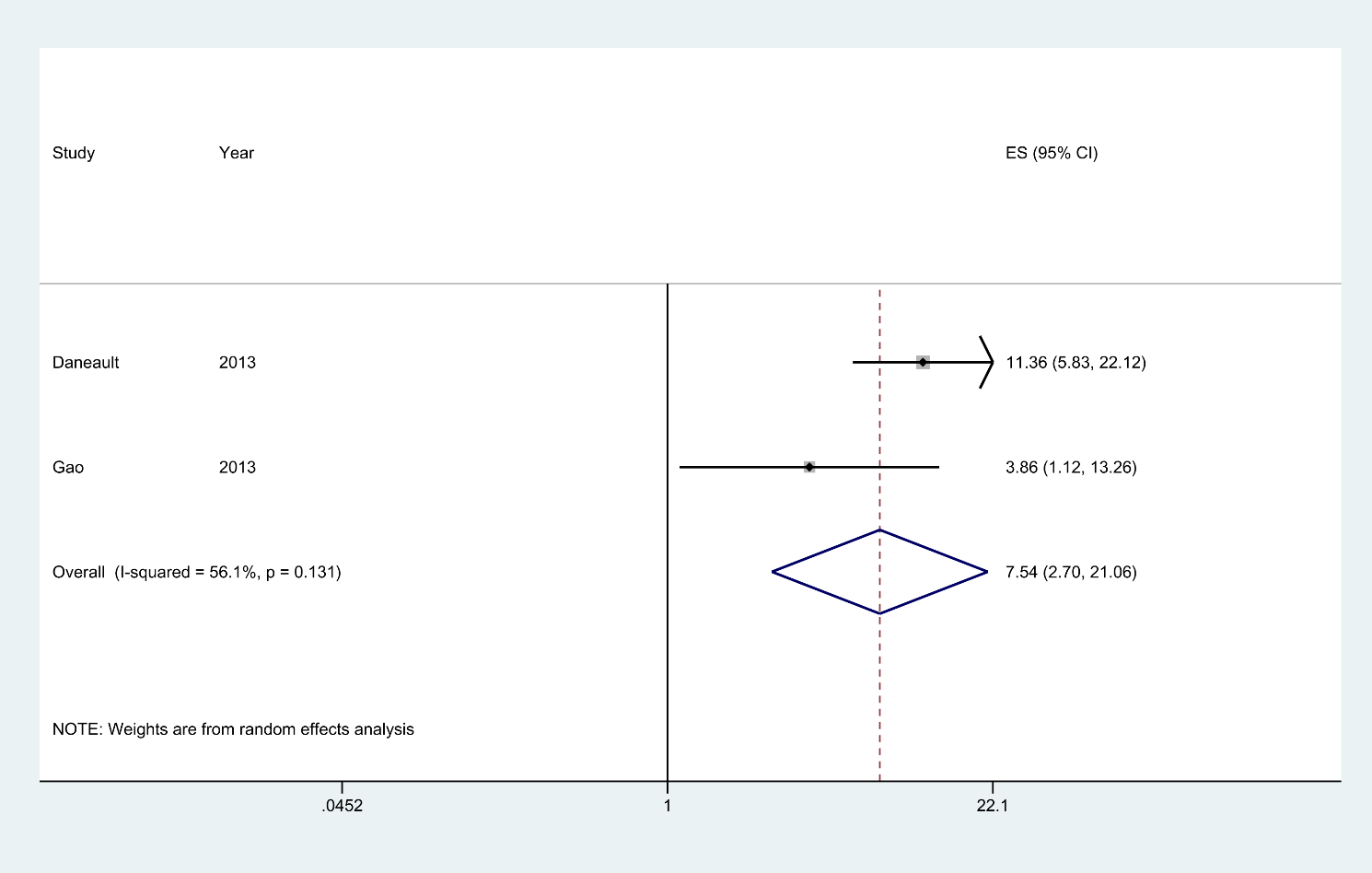


**Supplementary Fig. 20** Forest plot of hazard ratio of 1-year cardiac mortality among patients undergoing percutaneous coronary intervention (PCI), left ventricular ejection fraction (LVEF) under 40% vs above 40%


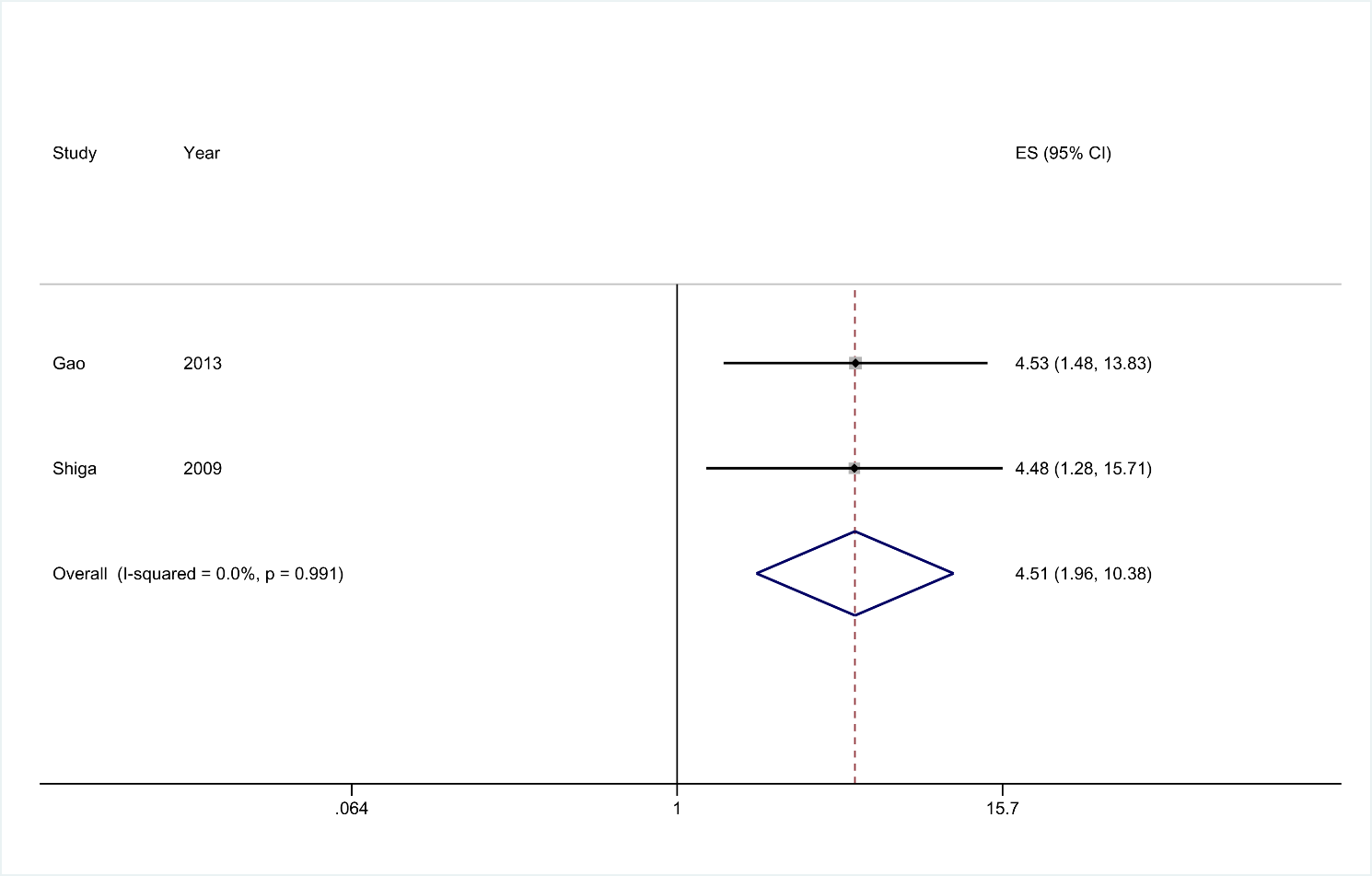


**Supplementary Fig. 21** Forest plot of hazard ratio of long-term cardiac mortality among patients undergoing percutaneous coronary intervention (PCI), left ventricular ejection fraction (LVEF) under 40% vs above 40%


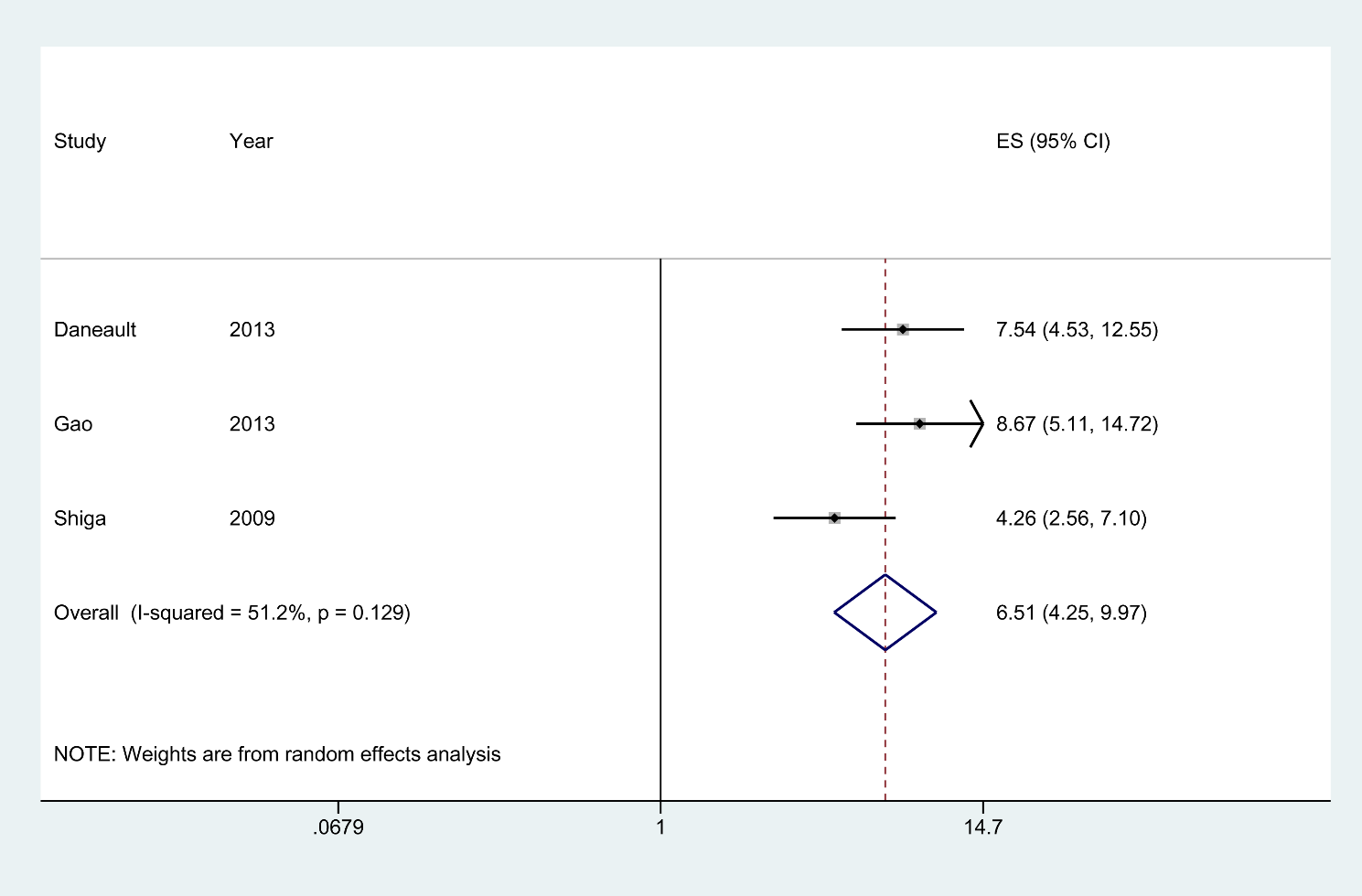


**Supplementary Fig. 22** Forest plot of hazard ratio of all-cause mortality among patients undergoing CTO percutaneous coronary intervention (PCI), left ventricular ejection fraction (LVEF) abnormal vs normal


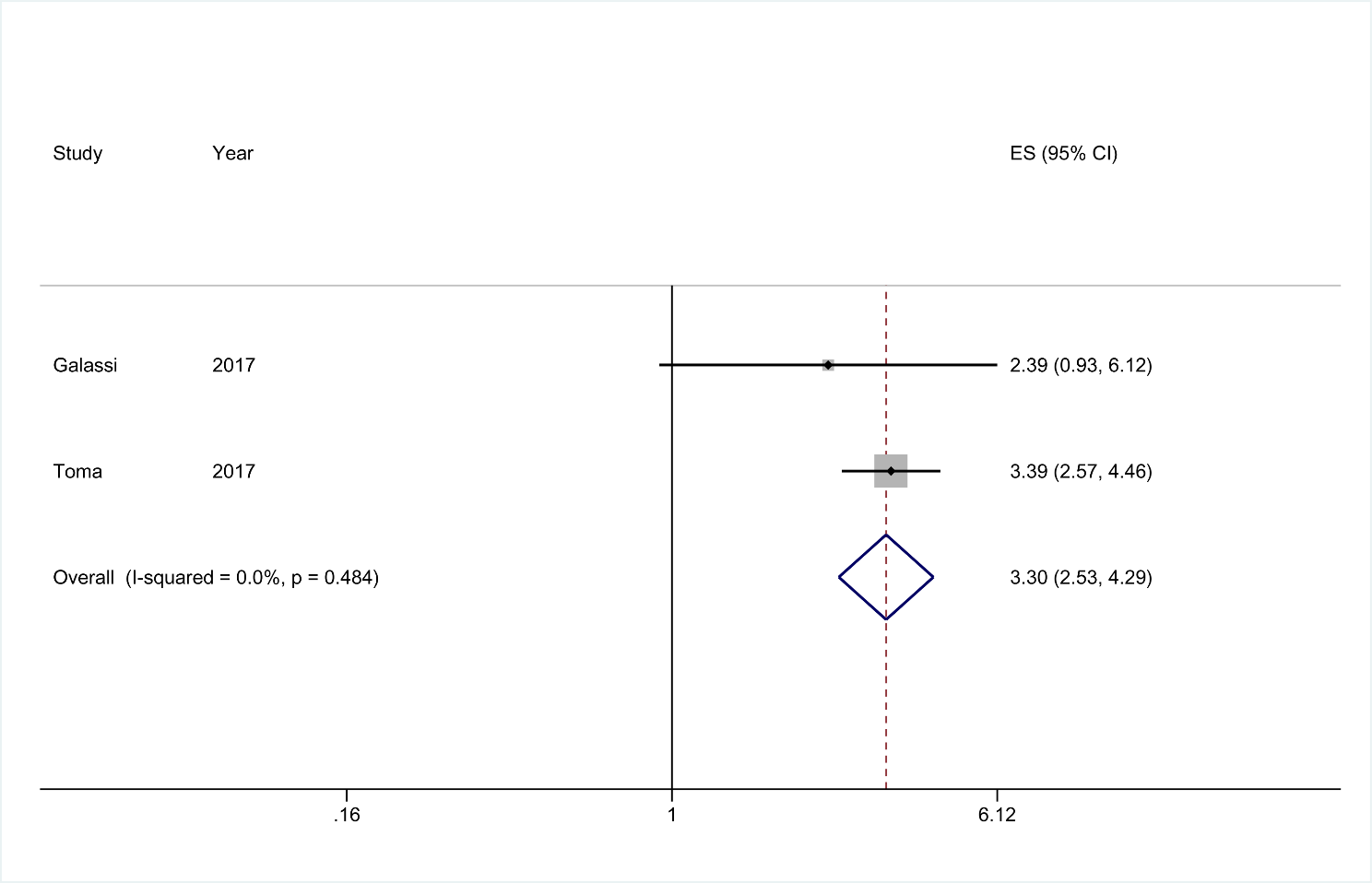


**Supplementary Fig. 23** Forest plot of odds ratio of major adverse cardiac events (MACE) among patients undergoing CTO percutaneous coronary intervention (PCI), left ventricular ejection fraction (LVEF) abnormal vs normal

**
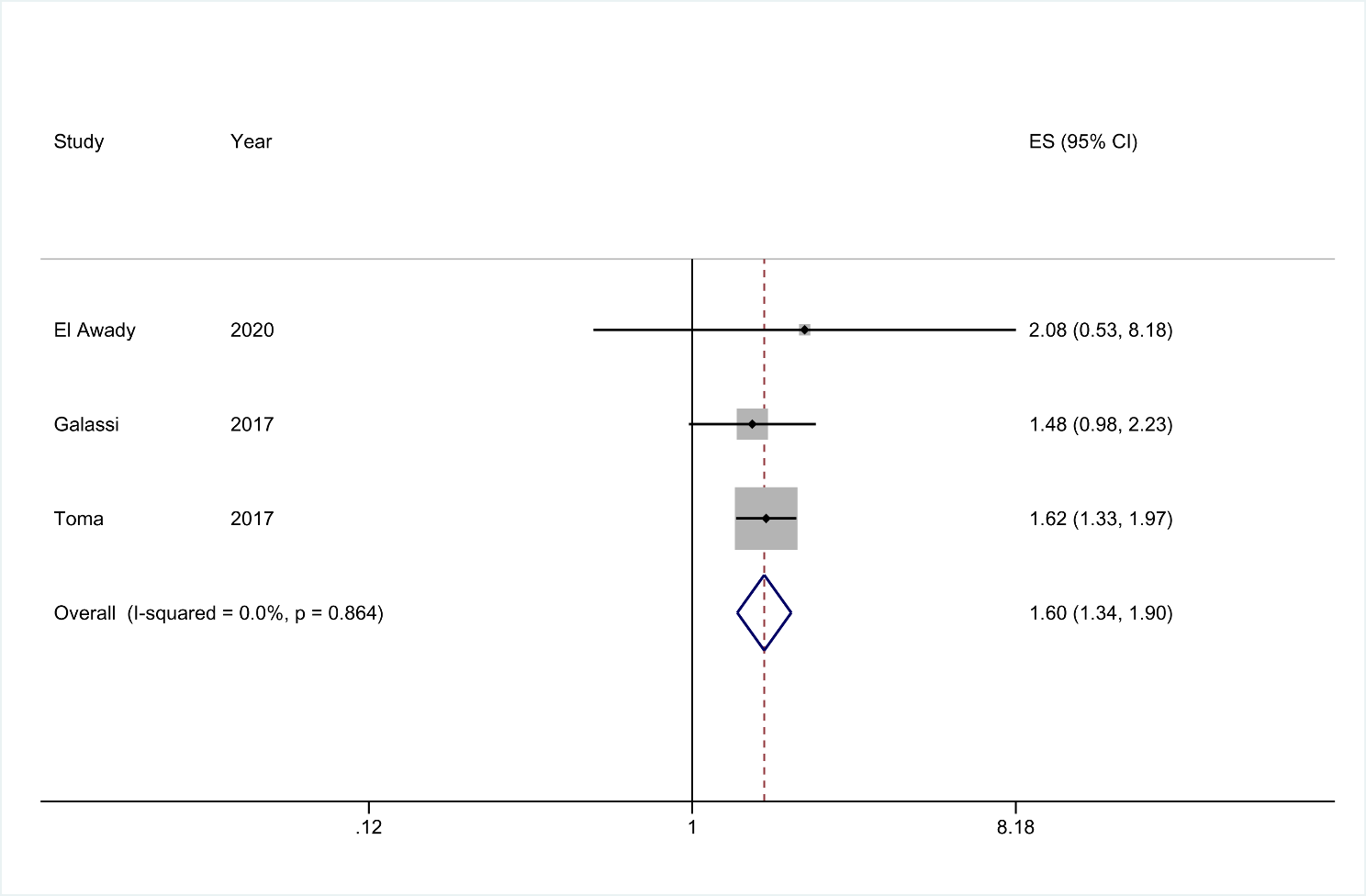
**

**Supplementary Fig. 24** Forest plot of hazard ratio of 30-day all-cause mortality among patients undergoing ST-elevation myocardial infarction (STEMI) percutaneous coronary intervention (PCI), left ventricular ejection fraction (LVEF) abnormal vs normal


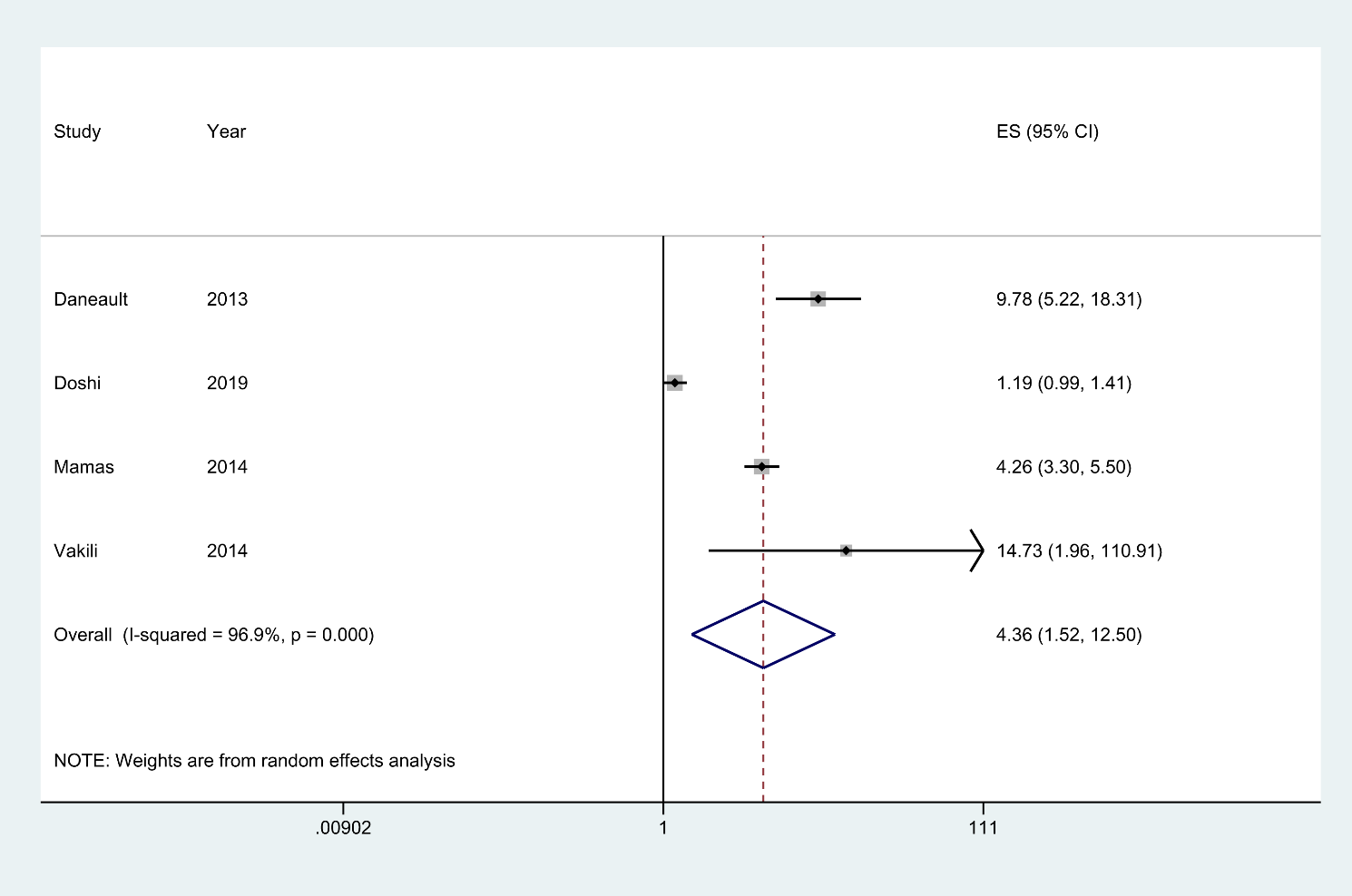


**Supplementary Fig. 25** Forest plot of hazard ratio of 1-year all-cause mortality among patients undergoing ST-elevation myocardial infarction (STEMI) percutaneous coronary intervention (PCI), left ventricular ejection fraction (LVEF) abnormal vs normal


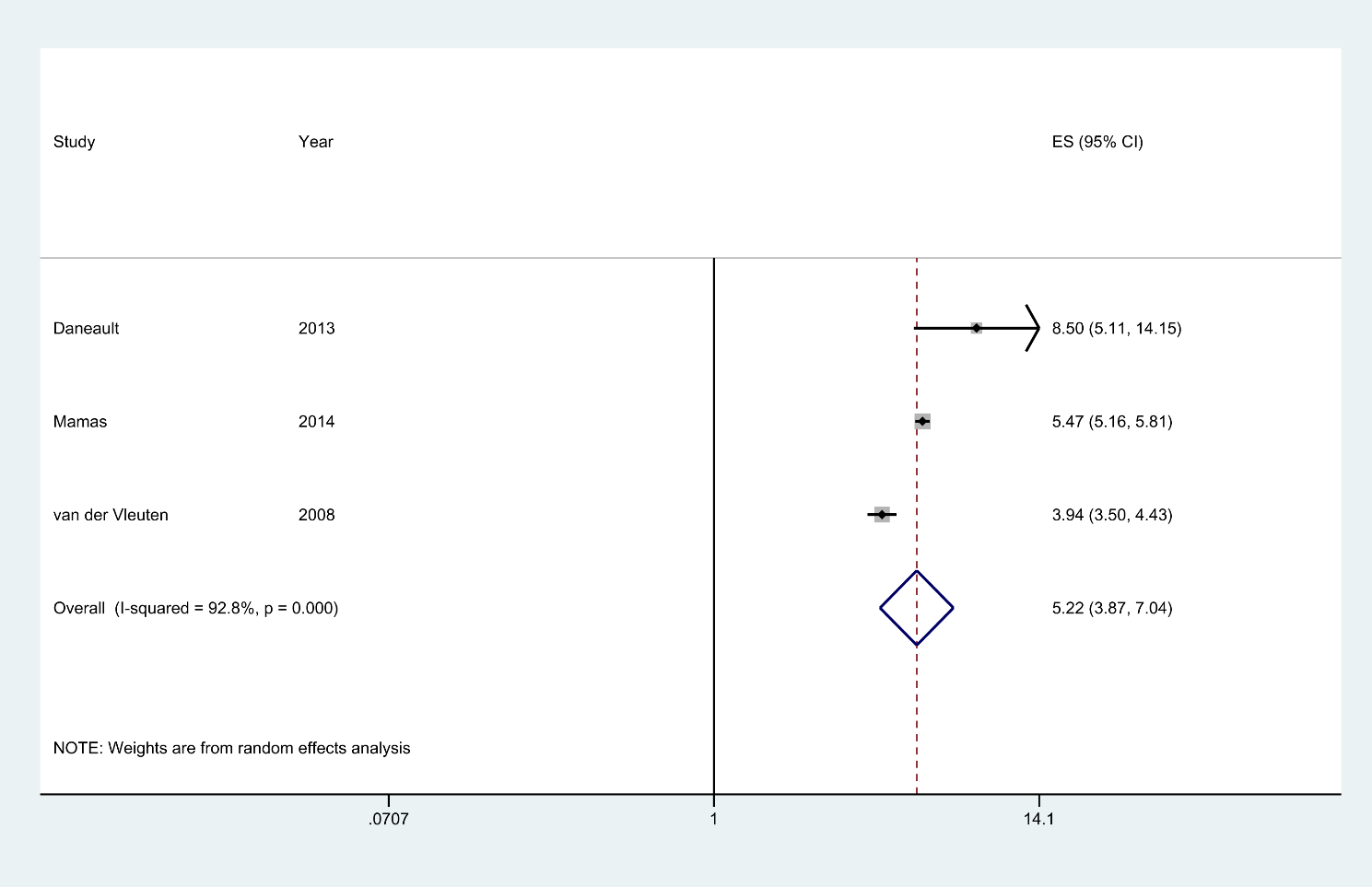


**Supplementary Fig. 26** Forest plot of hazard ratio of long-term all-cause mortality among patients undergoing ST-elevation myocardial infarction (STEMI) percutaneous coronary intervention (PCI), left ventricular ejection fraction (LVEF) abnormal vs normal


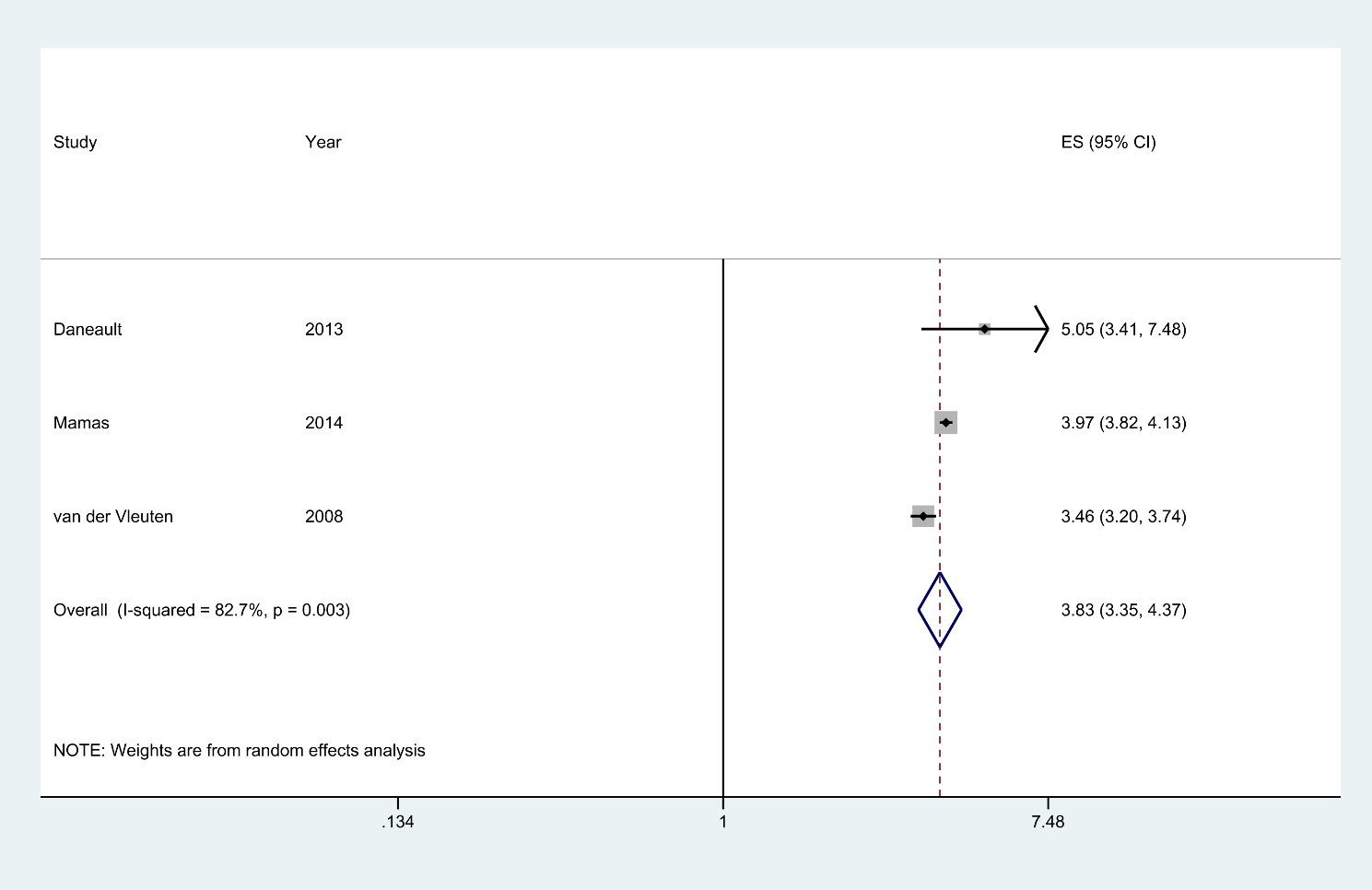


**Supplementary Fig. 27** Forest plot of odds ratio of 30-day major adverse cardiac events (major adverse cardiac events (MACE)) among patients undergoing ST-elevation myocardial infarction (STEMI) percutaneous coronary intervention (PCI), left ventricular ejection fraction (LVEF) abnormal vs normal


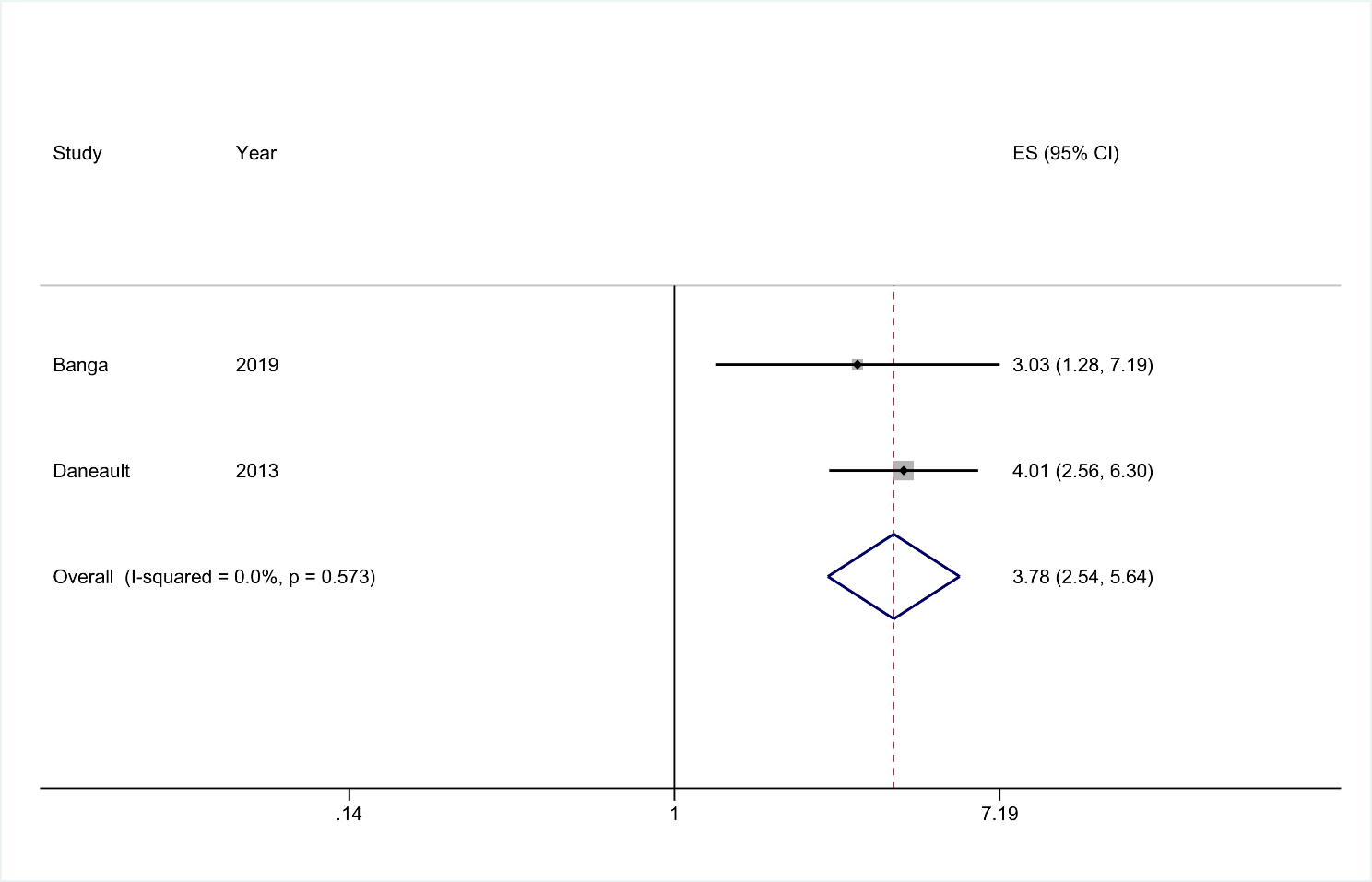


**Supplementary Fig. 28** Forest plot of hazard ratio of 30-day all-cause mortality among patients with heart failure undergoing percutaneous coronary intervention (PCI), heart failure with reduced ejection fraction vs heart failure with preserved ejection fraction


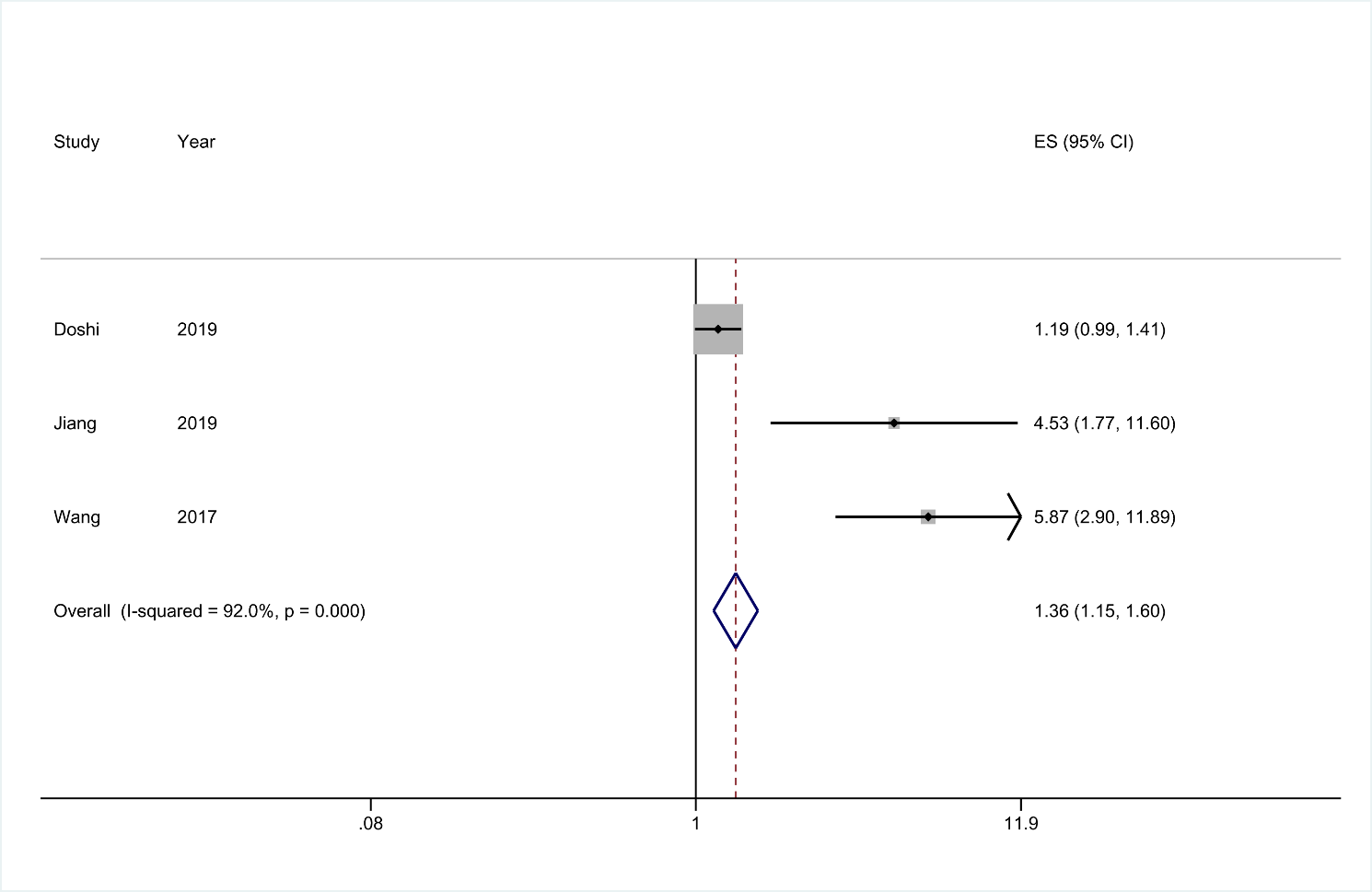


**Supplementary Fig. 29** Funnel plot of hazard ratio of 30-day all-cause mortality among patients undergoing percutaneous coronary intervention (PCI), left ventricular ejection fraction (LVEF) abnormal vs normal


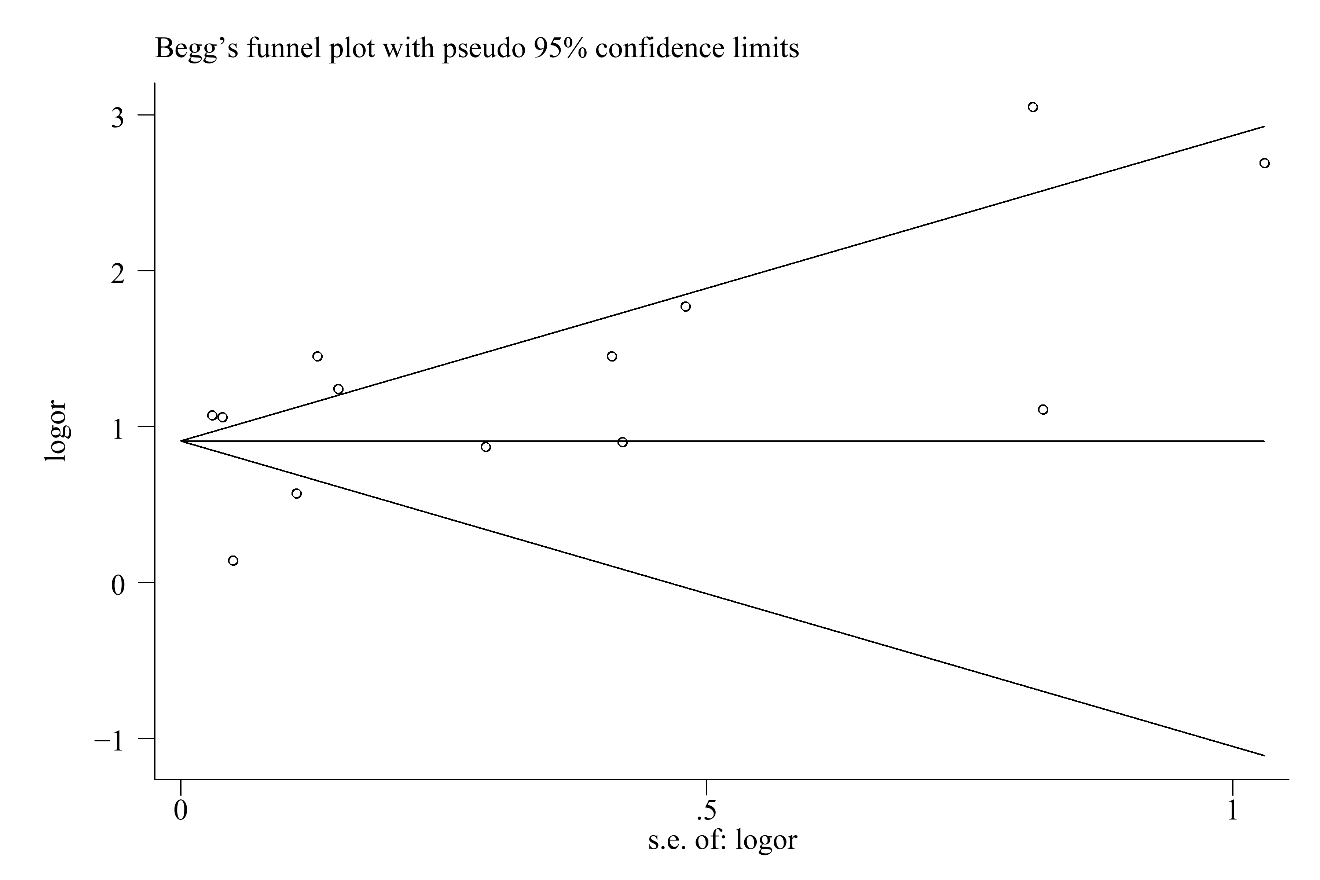


**References**

[1] Alidoosti M, Salarifar M, Zeinali AM, Kassaian SE, Dehkordi MR, Fatollahi MS. Short- and long-term outcomes of percutaneous coronary intervention in patients with low, intermediate and high ejection fraction. Cardiovascular journal of Africa*.* 2008; 19: 17-21.

[2] Banga S, Gumm DC, Kizhakekuttu TJ, Emani VK, Singh S, Singh S*, et al.* Left Ventricular Ejection Fraction along with Zwolle Risk Score for Risk Stratification to Enhance Safe and Early Discharge in STEMI Patients Undergoing Primary Percutaneous Coronary Intervention: A Retrospective Observational Study. Cureus*.* 2019; 11: e5272.

[3] Daneault B, Généreux P, Kirtane AJ, Witzenbichler B, Guagliumi G, Paradis JM*, et al.* Comparison of Three-year outcomes after primary percutaneous coronary intervention in patients with left ventricular ejection fraction <40% versus ≥ 40% (from the HORIZONS-AMI trial). The American journal of cardiology*.* 2013; 111: 12-20.

[4] Doshi R, Patel K, Gupta N, Gupta R, Meraj P. Characteristics and in-hospital outcomes of hospitalisations with heart failure with reduced or preserved ejection fraction undergoing percutaneous coronary intervention. Irish journal of medical science*.* 2019; 188: 791-799.

[5] El Awady WS, Samy M, Al-Daydamony MM, Abd El Samei MM, Shokry KAEA. Periprocedural and clinical outcomes of percutaneous coronary intervention of chronic total occlusions in patients with low- and mid-range ejection fractions. Egyptian Heart Journal*.* 2020; 72: 28.

[6] Galassi AR, Boukhris M, Toma A, Elhadj Z, Laroussi L, Gaemperli O*, et al.* Percutaneous Coronary Intervention of Chronic Total Occlusions in Patients With Low Left Ventricular Ejection Fraction. JACC. Cardiovascular interventions*.* 2017; 10: 2158-2170.

[7] Holper EM, Blair J, Selzer F, Detre KM, Jacobs AK, Williams DO*, et al.* The impact of ejection fraction on outcomes after percutaneous coronary intervention in patients with congestive heart failure: an analysis of the National Heart, Lung, and Blood Institute Percutaneous Transluminal Coronary Angioplasty Registry and Dynamic Registry. American heart journal*.* 2006; 151: 69-75.

[8] Jiang L, Song Y, Xu JJ, Tang XF, Wang HH, Jiang P*, et al.* [Outcome of patients with coronary artery disease and left ventricular ejection fraction less than 50% undergoing percutaneous coronary intervention]. Zhonghua xin xue guan bing za zhi*.* 2017; 45: 1058-1066.

[9] Jiang Y, Hu S, Cao M, Li X, Zhou J, Ding B*, et al.* Evaluation of acute myocardial infarction patients with mid-range ejection fraction after emergency percutaneous coronary intervention. Postgraduate medical journal*.* 2019; 95: 355-360.

[10] Marui A, Kimura T, Nishiwaki N, Mitsudo K, Komiya T, Hanyu M*, et al.* Comparison of five-year outcomes of coronary artery bypass grafting versus percutaneous coronary intervention in patients with left ventricular ejection fractions 50% versus >50% (from the CREDO-Kyoto PCI/CABG registry cohort-2). American Journal of Cardiology*.* 2014; 114: 988-996.

[11] Sardi GL, Gaglia MA, Jr., Maluenda G, Torguson R, Laynez-Carnicero A, Ben-Dor I*, et al.* Outcome of percutaneous coronary intervention utilizing drug-eluting stents in patients with reduced left ventricular ejection fraction. The American journal of cardiology*.* 2012; 109: 344-351.

[12] Shiga T, Hagiwara N, Ogawa H, Takagi A, Nagashima M, Yamauchi T*, et al.* Sudden cardiac death and left ventricular ejection fraction during long-term follow-up after acute myocardial infarction in the primary percutaneous coronary intervention era: results from the HIJAMI-II registry. Heart (British Cardiac Society)*.* 2009; 95: 216-220.

[13] Son YJ, Shim SK, Hwang SY, Ahn JH, Yu HY. Impact of left ventricular ejection fraction and medication adherence on major adverse cardiac events during the first year after successful primary percutaneous coronary interventions. Journal of Clinical Nursing*.* 2016; : .

[14] Sutton NR, Li S, Thomas L, Wang TY, De Lemos JA, Enriquez JR*, et al.* The association of left ventricular ejection fraction with clinical outcomes after myocardial infarction: Findings from the Acute Coronary Treatment and Intervention Outcomes Network (ACTION) Registry-Get with the Guidelines (GWTG) Medicare-linked database. American heart journal*.* 2016; 178: 65-73..

[15] Toma A, Stähli BE, Gick M, Gebhard C, Kaufmann BA, Mashayekhi K*, et al.* Comparison of Benefit of Successful Percutaneous Coronary Intervention for Chronic Total Occlusion in Patients With Versus Without Reduced (≤40%) Left Ventricular Ejection Fraction. The American journal of cardiology*.* 2017; 120: 1780-1786.

[16] Vakili H, Sadeghi R, Rezapoor P, Gachkar L. In-hospital outcomes after primary percutaneous coronary intervention according to left ventricular ejection fraction. ARYA atherosclerosis*.* 2014; 10: 211-217.

[17] Wang K, Li HL, Bei WJ, Gguo XS, Chen SQ, Mohammed S*, et al.* Association of left ventricular ejection fraction with contrast-induced nephropathy and mortality following coronary angiography or intervention in patients with heart failure. Therapeutics and Clinical Risk Management*.* 2017; 13: 887-895.

[18] Ye Z, Lu H, Li L. Reduced Left Ventricular Ejection Fraction Is a Risk Factor for In-Hospital Mortality in Patients after Percutaneous Coronary Intervention: A Hospital-Based Survey. BioMed research international*.* 2018; 2018: 8753176.

[19] Zhong J, Chen Q, Chen L, Ye Z, Chen H, Sun J*, et al.* Physiological benefits evaluated by quantitative flow ratio in patients with reduced left ventricular ejection fraction who underwent percutaneous coronary intervention. BMC cardiovascular disorders*.* 2020; 20: 523.

[20] Alaswad K, Basir MB, Khandelwal A, Schreiber T, Lombardi W, O'Neill W. The Role of Mechanical Circulatory Support During Percutaneous Coronary Intervention in Patients Without Severely Depressed Left Ventricular Function. The American journal of cardiology*.* 2018; 121: 703-708.

[21] Biondi-Zoccai G, Sheiban I, Moretti C, Palmerini T, Marzocchi A, Capodanno D*, et al.* Appraising the impact of left ventricular ejection fraction on outcomes of percutaneous drug-eluting stenting for unprotected left main disease: insights from a multicenter registry of 975 patients. Clinical research in cardiology : official journal of the German Cardiac Society*.* 2011; 100: 403-411.

[22] De Silva K, Webb I, Sicard P, Lockie T, Pattinson S, Redwood S*, et al.* Does left ventricular function continue to influence mortality following contemporary percutaneous coronary intervention? Coronary artery disease*.* 2012; 23: 155-161.

[23] Gao Z, Xu B, Kirtane AJ, Yang YJ, Yuan JQ, Chen JL*, et al.* Impact of depressed left ventricular function on outcomes in patients with three-vessel coronary disease undergoing percutaneous coronary intervention. Chinese medical journal*.* 2013; 126: 609-614.

[24] Halkin A, Stone GW, Dixon SR, Grines CL, Tcheng JE, Cox DA*, et al.* Impact and determinants of left ventricular function in patients undergoing primary percutaneous coronary intervention in acute myocardial infarction. The American journal of cardiology*.* 2005; 96: 325-331.

[25] Jackson M, Austin D, Kwok CS, Rashid M, Kontopantelis E, Ludman P*, et al.* The impact of diabetes on the prognostic value of left ventricular function following percutaneous coronary intervention: Insights from the British Cardiovascular Intervention Society. Catheterization and cardiovascular interventions : official journal of the Society for Cardiac Angiography & Interventions*.* 2018; 92: E393-e402.

[26] Keelan PC, Johnston JM, Koru-Sengul T, Detre KM, Williams DO, Slater J*, et al.* Comparison of In-Hospital and One-Year outcomes in patients with left ventricular ejection fractions ≤40%, 41% to 49%, and ≥50% having percutaneous coronary revascularization. The American journal of cardiology*.* 2003; 91: 1168-1172.

[27] Kwok CS, Anderson SG, McAllister KS, Sperrin M, O'Kane PD, Keavney B*, et al.* Impact of age on the prognostic value of left ventricular function in relation to procedural outcomes following percutaneous coronary intervention: insights from the British Cardiovascular Intervention Society. Catheterization and cardiovascular interventions : official journal of the Society for Cardiac Angiography & Interventions*.* 2015; 85: 944-951.

[28] Levi A, Bental T, Assali AR, Lev EI, Vaknin Assa H, Shaul AA*, et al.* Dynamic changes in left ventricular function after a percutaneous coronary intervention: prevalence, predictors, and prognosis. Coronary artery disease*.* 2016; 27: 199-206.

[29] Mamas MA, Anderson SG, O'Kane PD, Keavney B, Nolan J, Oldroyd KG*, et al.* Impact of left ventricular function in relation to procedural outcomes following percutaneous coronary intervention: insights from the British Cardiovascular Intervention Society. European heart journal*.* 2014; 35: 3004-3012a.

[30] Marsico F, Morenghi E, Parenti DZ, Milone F, Maiello L, Carcagnì A*, et al.* Immediate and late results of coronary angioplasty in patients with severe left ventricular dysfunction. Italian heart journal : official journal of the Italian Federation of Cardiology*.* 2003; 4: 838-842.

[31] Singh M, Rihal CS, Lennon RJ, Spertus J, Rumsfeld JS, Holmes DR. Bedside Estimation of Risk From Percutaneous Coronary Intervention: The New Mayo Clinic Risk Scores. Mayo Clinic Proceedings*.* 2007; 82: 701-708.

[32] van der Vleuten PA, Rasoul S, Huurnink W, van der Horst IC, Slart RH, Reiffers S*, et al.* The importance of left ventricular function for long-term outcome after primary percutaneous coronary intervention. BMC cardiovascular disorders*.* 2008; 8: 4.

[33] Wallace TW, Berger JS, Wang A, Velazquez EJ, Brown DL. Impact of Left Ventricular Dysfunction on Hospital Mortality Among Patients Undergoing Elective Percutaneous Coronary Intervention. The American journal of cardiology*.* 2009; 103: 355-360.
